# Benzene and Naphthalene Degrading Bacterial Communities in an Oil Sands Tailings Pond

**DOI:** 10.3389/fmicb.2017.01845

**Published:** 2017-09-28

**Authors:** Fauziah F. Rochman, Andriy Sheremet, Ivica Tamas, Alireza Saidi-Mehrabad, Joong-Jae Kim, Xiaoli Dong, Christoph W. Sensen, Lisa M. Gieg, Peter F. Dunfield

**Affiliations:** ^1^Department of Biological Sciences, University of Calgary, Calgary, AB, Canada; ^2^Department of Biology and Ecology, Faculty of Sciences, University of Novi Sad, Novi Sad, Serbia; ^3^Department of Biological Sciences, University of Alberta, Edmonton, AB, Canada; ^4^Department of Biochemistry and Molecular Biology in the Cumming School of Medicine, University of Calgary, Calgary, AB, Canada; ^5^Department of Geoscience, University of Calgary, Calgary, AB, Canada; ^6^Institute of Computational Biotechnology, Graz University of Technology, Graz, Austria

**Keywords:** oil sands, tailings pond, hydrocarbon degradation, benzene, naphthalene, metagenomics, stable isotope probing

## Abstract

Oil sands process-affected water (OSPW), produced by surface-mining of oil sands in Canada, is alkaline and contains high concentrations of salts, metals, naphthenic acids, and polycyclic aromatic compounds (PAHs). Residual hydrocarbon biodegradation occurs naturally, but little is known about the hydrocarbon-degrading microbial communities present in OSPW. In this study, aerobic oxidation of benzene and naphthalene in the surface layer of an oil sands tailings pond were measured. The potential oxidation rates were 4.3 μmol L^−1^ OSPW d^−1^ for benzene and 21.4 μmol L^−1^ OSPW d^−1^ for naphthalene. To identify benzene and naphthalene-degrading microbial communities, metagenomics was combined with stable isotope probing (SIP), high-throughput sequencing of 16S rRNA gene amplicons, and isolation of microbial strains. SIP using ^13^C-benzene and ^13^C-naphthalene detected strains of the genera *Methyloversatilis* and *Zavarzinia* as the main benzene degraders, while strains belonging to the family *Chromatiaceae* and the genus *Thauera* were the main naphthalene degraders. Metagenomic analysis revealed a diversity of genes encoding oxygenases active against aromatic compounds. Although these genes apparently belonged to many phylogenetically diverse taxa, only a few of these taxa were predominant in the SIP experiments. This suggested that many members of the community are adapted to consuming other aromatic compounds, or are active only under specific conditions. 16S rRNA gene sequence datasets have been submitted to the Sequence Read Archive (SRA) under accession number SRP109130. The Gold Study and Project submission ID number in Joint Genome Institute IMG/M for the metagenome is Gs0047444 and Gp0055765.

## Introduction

The Athabasca oil sands reserves, located in northern Alberta, Canada, contribute more than 50% of the total crude oil production in Canada (National Energy Board, 2015). It is estimated that bitumen production could reach 5–6 million barrels per day by 2050 if demand persists (Rahnama et al., 2013), although the availability of shale oil and alternative fuels may depress this demand. For every cubic meter of bitumen extracted, 4 m^3^ of fluid tailings are produced. This consists of oil sands process-affected water (OSPW), sand, clays, residual bitumen and dissolved inorganic and organic compounds (Holowenko et al., 2002; Quagraine et al., 2005). Fluid tailings are deposited into open ponds to allow settling of particles, reuse of surface water for extraction, and long-term pollutant containment for on-site reclamation (Government of Alberta, 2009).

In general, OSPW is alkaline (pH 7.8–8) with high contents of salts (2.2 g L^−1^), metals, sulfides, naphthenic acids (NAs), and polycyclic aromatic compounds (PAHs) (Quagraine et al., 2005; Kelly et al., 2009, 2010; Saidi-Mehrabad et al., 2013). Some of the organic contaminants are derived from the naphtha (usually a mixture of C_3_–C_14_ alkanes, BTEX, and iso-paraffins) used as a diluent in processing of bitumen, and others are derived from bitumen (Siddique et al., 2007). The NAs and PAHs are particular compounds of concern (Rogers et al., 2002; Quagraine et al., 2005; Allen, 2008; Wayland et al., 2008; Grewer et al., 2010), and questions have been raised about the potential movement of these compounds from tailings ponds into surrounding environments (Wayland et al., 2008). PAHs and heterocyclic aromatic compounds account for 0.01–10 mg kg^−1^ of fine tails in Alberta tailings ponds (Fine Tailings Fundamentals Consortium (FTFC), 1995; Wayland et al., 2008). Tailings ponds are predominantly anoxic, except for a (roughly 1-m-deep) surface water cap that is partially oxygenated and supports some aerobic microbial activities such as methane oxidation (Saidi-Mehrabad et al., 2013). Previous studies on biodegradation of compounds of concern in tailings ponds have therefore concentrated on anoxic conditions. Relatively little is known about activities in the more oxic surface water (Foght et al., 2017).

The major aerobic degradation pathways for both saturated and aromatic hydrocarbons involve the addition of oxygen atoms in reactions carried out by oxygenase enzymes (Atlas, 1981). Genes encoding dioxygenases, monooxygenases, and extradiol ring-cleavage enzymes were abundant in metagenomes constructed from surface water samples of oil sands tailings ponds (An et al., 2013; Saidi-Mehrabad et al., 2013), showing a diversity of putative hydrocarbon degradation mechanisms. Many of the bacterial genera detected in the surface water are known to have potential for aerobic hydrocarbon degradation, including *Brevundimonas* (*Caulobacterales*), *Methylocaldum* (*Methylococcales*); *Xanthobacter* (*Rhizobiales*), *Flavobacterium* (*Flaviobacteriales*), and diverse members of the order *Burkholderiales* (An et al., 2013; Saidi-Mehrabad et al., 2013). Aerobic microbial communities in oil sands tailings ponds therefore may have potential in bioremediation (Golby et al., 2012; Saidi-Mehrabad et al., 2013).

The importance of aerobic hydrocarbon degradation has been demonstrated in many environments (DeLaune et al., 1980; Atlas, 1991; Das and Chandran, 2011). Aerobic microbial populations are known to be pivotal in the reclamation of industrially affected waters (Oller et al., 2011; Tocchi et al., 2012). Although oil sands tailings ponds are largely anoxic at present, understanding aerobic degradation processes is valuable because some of the reclamation strategies proposed for them involve increasing the depth of the oxic water cap. The “wet-landscape” approach proposes the conversion of tailings ponds to End-Pit Lakes or other wetlands (Allen, 2008; Wayland et al., 2008). In an End-Pit Lake strategy, freshwater (and OSPW) is used to form a deeper water cap on a tailings pond, in order to increase O_2_ supply (thereby accelerating biodegradation), provide seed microbes and algae, and dilute the contaminants present (Allen, 2008; Wayland et al., 2008). It is expected that tailings will settle, density, release pore water into the water column, and detoxify naturally over time.

In this study, we combined metagenomics, Stable Isotope Probing (SIP), high-throughput sequencing of 16S rRNA gene amplicons, and cultivation to examine bacterial communities responsible for the degradation of two model aromatic hydrocarbons, benzene and naphthalene, in the oxic surface OSPW layer of an oil sands tailings pond.

## Materials and methods

### Sampling and water chemistry

The West-In-Pit (WIP) of Syncrude Canada, Ltd, established in 1997, was primarily used as a storage facility for fluid fine tailings and recycle water supply before its closure in December 2012 and repurposing as the first model End-Pit Lake for the oil sands industry in Canada (Syncrude, 2012). Before closure, the pond stored approximately 200 Mm^3^ of fluid fine tailings, capped with a minimum 5-m-deep layer (30 Mm^3^) of OSPW. Tailings porewater from this operation has previously been noted to contain 2,600 ng L^−1^ of total PAH, including 101 ng L^−1^ (780 nM) naphthalene (Madill et al., 1999). The OSPW used in this study was surface water (0–10 cm) sampled in August 2011. The chemical composition of the OSPW and the water sampling methods were described previously (Saidi-Mehrabad et al., 2013). Throughout this article, OSPW sampled from the WIP tailings pond is termed WIP-OSPW.

### Potential hydrocarbon oxidation rates

Potential rates of benzene and naphthalene oxidation (i.e., rates with excess substrate added) were measured in 20 mL (benzene) or 40 mL (naphthalene) amounts of OSPW in 120-mL serum vials, which were sealed with butyl rubber stoppers (20 mm). Vials containing MilliQ water were used as abiotic controls, and vials containing unamended OSPW were used as controls with no substrate addition. There is a small amount of hydrocarbon and other substrates in OSPW, therefore a slow CO_2_ increase is expected. Hydrocarbon-amended samples, unfiltered OSPW controls, and MilliQ water controls were run in triplicates.

For benzene oxidation, 1 μl (= 11 μmol) of benzene (Sigma-Aldrich, St Louis, MO, USA) was added to 20 mL OSPW. Using a Henry's law constant of 0.18 M atm^−1^, the initial benzene concentration was 0.30 mM in the liquid phase and 0.075 mmol L^−1^ in the gas phase. For naphthalene degradation, a 4.0 mM naphthalene (Sigma-Aldrich) stock was prepared in a 20-mL inert non-degradable carrier 2, 2, 4, 4, 6, 8, 8-heptamethylnonane (HMN; Sigma-Aldrich) and 10-mL aliquots were added to 40-mL OSPW sample volumes. The concentration of naphthalene in HMN is well above the saturation point of naphthalene in water (about 0.24 mM), and therefore it is assumed that naphthalene is saturating, and that calculations of naphthalene degradation in the system need only account for the HMN phase.

Bottles were incubated at 20°C on a rotary shaker at 180 rpm to ensure aeration, for 23 days (benzene) or 14 days (naphthalene). At regular intervals, benzene and naphthalene were measured by gas chromatography as previously described (Berdugo-Clavijo et al., 2012; Fowler et al., 2012). Oxidation rates were calculated by linear regression of benzene and naphthalene depletion over time. Headspace CO_2_ and O_2_ were monitored by injection of gas samples into a Varian 450-GC gas chromatograph equipped with a thermal conductivity detector (150°C), after separation in a 2 mm × 0.5 m Hayesep N and a 2 mm × 1.2 m Molecular Sieve 16X column in series (70°C). CO_2_ production rates were calculated by linear regression of CO_2_ production vs. time.

### Stable isotope probing (SIP)

To identify microbial communities actively assimilating the model aromatic compounds, isotopically fully-labeled benzene (^13^C_6_H_6_ 99 atom%, Sigma-Aldrich) and naphthalene (^13^C_10_H_8_ 99 atom%) were used in DNA-SIP. To concentrate bacteria, 150 mL of OSPW were filtered through a 0.2-μm polysulfone filter (Pall Life Sciences, East Hills, NY, USA). The filter and 20 mL of OSPW were added to 100-mL serum vials and supplemented with 5 μM of labeled benzene or naphthalene. Vials containing 20 mL of filtered OSPW without added substrate were used as controls. Headspace CO_2_ was monitored for all SIP-enrichments and incubations were terminated when CO_2_ levels in the microcosms increased by around 1.0 μmol of CO_2_. After experimenting with different incubation times (3, 7, 9, and 14 days, data not shown), we chose the earliest times at which a shift toward heavy density fraction was visible in CsCl gradients, indicating ^13^C incorporation into microbial genomes. Shifts were visible after 7 days (naphthalene) and 9 days (benzene), of incubation. Cells were collected by centrifugation for 10 min at 10,000 × g. DNA was extracted, purified, separated by isopycnic centrifugation in CsCl, and fractionated as described previously (Sharp et al., 2012). DNA extracted from OSPW incubated for the same time without addition of substrates was used as a control to determine the expected position of unlabeled DNA in CsCl gradients. Heavy ^13^C-labeled DNA fractions of labeled samples were selected as shown in Supplementary Figure S1. The corresponding highest-density fractions of OSPW receiving the same treatments (OSPW-heavy), and unfractionated DNA from fresh OSPW (OSPW-control) were used as controls.

Amplification of 16S rRNA genes, sequencing on a Roche 454 GS FLX, and analysis using QIIME were all carried out as described previously (Sharp et al., 2014). Briefly, 16S rRNA genes were amplified from the gradient fractions using FLX Titanium amplicon primers 454T_RA_X and 454T_F containing the 16S rRNA gene targeted primers 926f and 1392r at their 3′- ends, along with adaptors necessary for the Roche Titanium chemistry (Sharp et al., 2014). The QIIME software platform was used to analyze the sequences (Caporaso et al., 2010). Low-quality sequences were removed based on a minimum quality score of 25, Operational Taxonomic Units (OTUs) clustered based on 97% sequence identity, chimeras removed via ChimeraSlayer, and sequences classified via BLAST against the Greengenes database. Taxonomic identifications were verified by manual BLAST of representative OTU sequences against the NCBI database.

### Metagenomics

Metagenomic DNA from OSPW was prepared and extracted as described previously (Saidi-Mehrabad et al., 2013). DNA concentration was determined with a Qubit Fluorometer using a Quant-iT dsDNA HS Assay Kit (Invitrogen, Carlsbad, CA, USA). The DNA (12 μg) was sequenced using a combination of Roche 454 GS FLX and paired-end Illumina HiSeq2000 platforms at the Genome Quebec and McGill University Innovation Centre, Montreal, Quebec. Quality control and assembly of metagenomic data was performed at the University of Calgary Visual Genomics Center, Calgary, Canada according to previous reports (Saidi-Mehrabad et al., 2013; Tan et al., 2013; Aguilar et al., 2016). Annotation was conducted by submission to the Joint Genome Institute IMG platform (Markowitz et al., 2012).

Unassembled read based analysis was performed on the Illumina HiSeq2000 output due to the higher sequencing depth, although similar results were obtained when 454 reads were used. Clipping of the Illumina adapters, quality-trimming and size-filtering for the raw reads was performed using a BBDuk tool (trimk = 27, trimq = 20; minlen = 80, http://jgi.doe.gov/data-and-tools/bbtools/). Curated databases for key genes encoding enzymes for aerobic benzene and naphthalene degradation were constructed in a two-step process. First, core datasets for each gene were created by retrieving DNA sequences from NCBI Gene database using Enzyme Commission numbers and gene names. Next, the core datasets were extended by querying their entries using BLASTN against the NCBI non-redundant and environmental samples databases (Coordinators, 2013). Sequences with nucleotide identities of 70% and above were added to the corresponding datasets. Processed metagenomic sequences were then queried against the reference datasets using BLASTN. Reads with minimum nucleotide sequence identities of 50% and alignment lengths of 75 bp were recruited. Custom python and bash scripts were created to run BLASTN, process the output, and quantify and recruit reads matching the specified criteria. Scripts are available at the GitHub repository (release v1.14.3, https://github.com/dunfieldlab/mg_wrapser). Recruited reads, corresponding to each dataset, were then queried by BLASTN against the NCBI non-redundant database to validate functional identity and processed with MEtaGenome ANalyzer (MEGAN v.5.11.3) for taxonomic assignment (Huson et al., 2007).

### Isolation of hydrocarbon degrading bacteria

OSPW samples were initially enriched for 14 days with either 5 mM of benzene or naphthalene added (99.9%, Sigma-Aldrich). To cultivate bacterial strains from these enrichments, both complex media targeting versatile chemotrophs and basal mineral salts media containing the model hydrocarbons as sole carbon and energy sources were used. Enriched OSPW was diluted 1 : 10 with MilliQ water and 100-μl aliquots spread on 20% strength R2A (20R2A) plates adjusted to pH 8 with NaOH, as well as on a modification of the basal mineral salts medium M10 (Reasoner and Geldreich, 1985). M10 was prepared as described previously (Heyer et al., 2002), except that agar was substituted with 1.5% phytagel (Sigma-Aldrich), and MilliQ water was substituted with 0.2-μm-filtered OSPW to reproduce the natural conditions of tailings water. We named these modified media containing filtered tailings water 20R2A-T and M10-T. To specifically grow naphthalene-degrading microorganisms, a 10-fold serial dilution of the initial enrichment was applied (1 mL enrichment sample and 9 mL MilliQ water). Dilutions up to (10^−10^) were plated onto solid medium M10-T prepared in 125-mL wide-mouth jars (I-Chem, VWR, Radnor, PA, USA). These growth vessels were inverted and 3 g of naphthalene placed on the lids to diffuse naphthalene vapor as previously described (Jeon et al., 2004). For benzene-degrading microorganisms, OSPW samples enriched with 5 mM benzene were serially diluted as above, spread onto 20R2A-T in wide-mouth jars, and incubated with the addition of 0.1 mL of benzene per jar at 20°C for 60–70 days.

Colonies were picked and streaked onto new plates of the same medium until pure colonies were obtained. Colony PCR was performed to screen the isolates. Single colonies were picked with autoclaved toothpicks and frozen at –80°C overnight in 0.2-mL tubes to lyse cells. 16S rRNA genes were amplified using the universal primer set 9f and 1492b (Weisburg et al., 1991). PCR reaction conditions were: initial denaturation at 94°C for 10 min, followed by 35 cycles of 1 min at 94°C, 1 min at 56°C and 2 min at 72°C, and a 10-min final elongation at 72°C. PCR products were visualized on a 1% agarose gel and purified with an EZ-10 Spin Column PCR Purification Kit (BioBasic Inc., Markham, ON, Canada). DNA concentration was determined as described above. Sanger sequencing was performed at the University of Calgary Core DNA Services using Applied Biosystems 3730xl (96 capillary) genetic analyzer (Applied Biosystems, Foster City, CA, USA). Forward and reverse sequences were merged via EMBOSS 6.3.1 merger (Néron et al., 2009) and >1,300 bp sequences were identified via BLASTN (Altschul et al., 1997) against the EzTaxon server (Kim et al., 2012).

A number of isolates were tested for their benzene degradation. Exponentially growing bacteria in 20R2A-T medium (pH 8.5) were transferred to 20R2A liquid medium (20 mL in a 100-mL serum vial), and sealed with butyl rubber stoppers. Benzene (6 μM) was added to the cultures. Each sample was run in triplicate. Loss of benzene was measured in duplicate pure cultures as described above. Growth was monitored by measuring OD_600_ on an Ultrospec 10 Cell Density Meter (Amersham Biosciences, Piscataway, NJ).

## Results

### Potential hydrocarbon oxidation rates

The benzene and naphthalene oxidation rates measured were roughly linear over time (Supplementary Figures S2, S3). Therefore we assume 0-order kinetics (i.e., the enzymes are saturated and the rates reported are V_max_ values not dependent on substrate concentration). The potential benzene oxidation rate was 4.3 μmol L^−1^ OSPW d^−1^ in the first 23 d, and the naphthalene oxidation rate was 21.4 μmol L^−1^ OSPW d^−1^ over 14 d (Supplementary Figures S2, S3). Conditions remained oxic throughout the incubations (the maximum O_2_ decline was from 21% to 17% v/v). In the benzene enrichment 5.6 μmol of CO_2_ L^−1^ OSPW d^−1^ was produced, 1.3 times of the expected CO_2_ production based on the reaction: C_6_H_6_ + 2.5O_2_ + NH_3_ → C_5_H_7_O_2_N + CO_2_ + H_2_O (Shuler and Kargi, 2002). In the naphthalene enriched samples, 144.1 μmol of CO_2_ L^−1^ OSPW d^−1^ was produced, or 1.3 times of the expected CO_2_ production based on the reaction: C_10_H_8_ + 7O_2_ + NH_3_ → C_5_H_7_O_2_N + 5CO_2_ + 2H_2_O (Shuler and Kargi, 2002). The slightly higher than expected CO_2_ production indicates that less C is assimilated from these compounds than predicted by simple models, or that added benzene and naphthalene enhance the degradation of other hydrocarbon substrates available in OSPW (Suthersan and McDonough, 1996).

### SIP and 16S rRNA gene sequencing

Density gradient separation of extracted DNA after 9 days (benzene) or 7 days (naphthalene) of incubation indicated that ^13^C-substrate incubations led to a small shift in the DNA distribution toward heavier densities (Supplementary Figure S1). The positions of the “heavy” DNA fractions representing the active ^13^C-assimilating communities were determined by comparing density gradients of the DNA extracted from the SIP incubations to that of the control samples, as indicated in Supplementary Figure S1.

**Figure 1:**
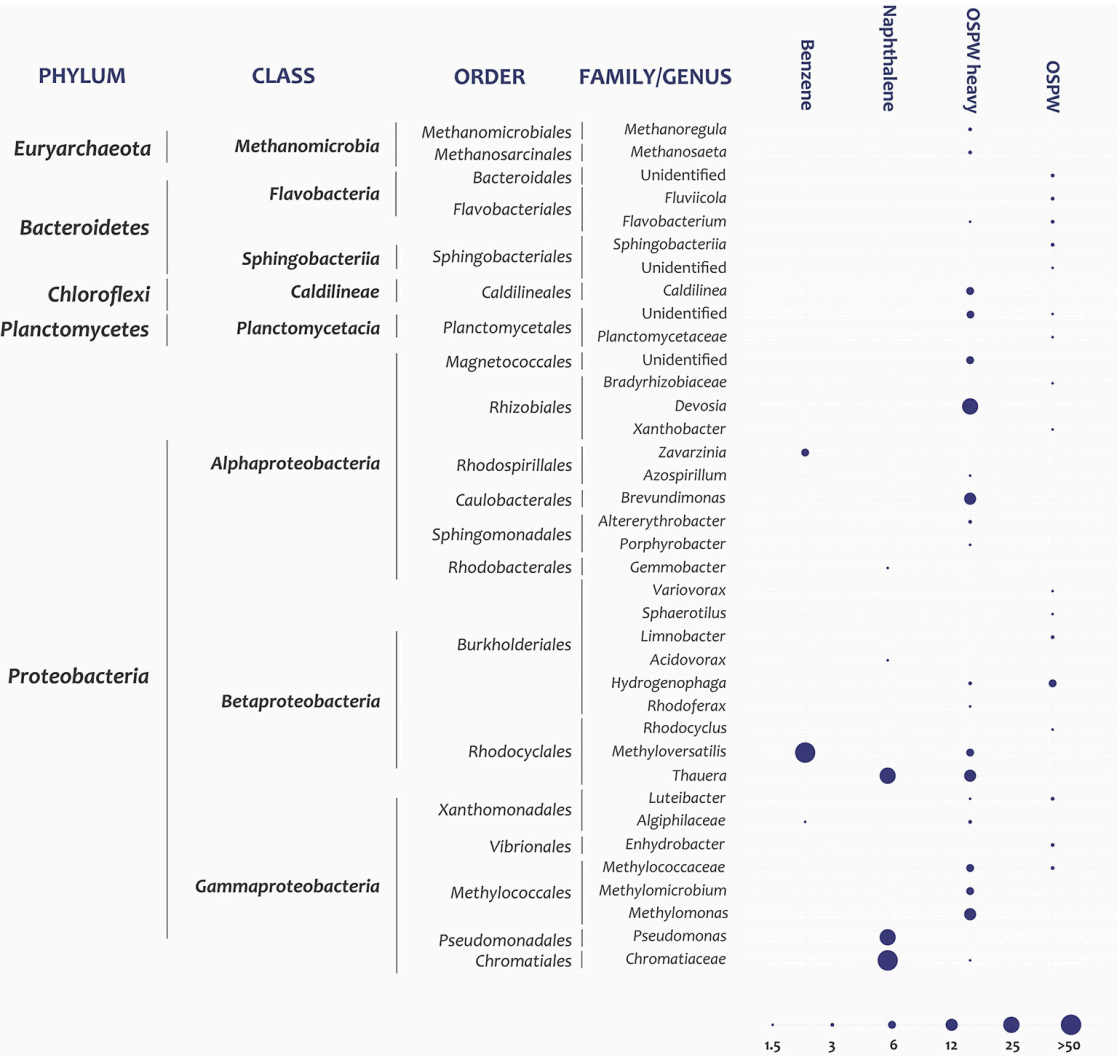
Predominant taxa detected in DNA of control OSPW samples, and in heavy DNA fractions extracted after incubation with ^13^C benzene or ^13^C naphthalene. Data are relative abundances of taxa within sequenced 16S rRNA gene amplicons (only OTUs >1% of the total reads are shown). The lowest taxonomic level confidently assigned is based on 16S rRNA identity thresholds defined by Yarza et al. (2014). Phylum, class, order, and either family or genus is indicated. Benzene: heavy-DNA fraction of benzene-amended OSPW incubated for 9 days; Naphthalene: heavy-DNA fraction of naphthalene- amended OSPW incubated for 7 days; OSPW-heavy: heavy fraction of OSPW incubated for the same amount of time as the amended samples; and OSPW-control: complete, unfractionated DNA from OSPW. The bubbles show 6 abundance classes (1–1.75%; 1.76–4.5%; 4.6–9%; 9.1–18.5%; 18.6–37.5%; >37.6%).

Community analysis of 16S rRNA genes amplified from OSPW DNA prior to the SIP incubations showed both methanogenic archaea and diverse bacteria. The combination of anaerobes, aerobes, and facultative anaerobes has been noted before (An et al., 2013; Saidi-Mehrabad et al., 2013) and is probably the result of active water circulation between anoxic and oxic water zones. The dominant classes/phyla of bacteria were *Betaproteobacteria, Alphaproteobacteria*, and *Flavobacteria* (Supplementary Figure S4). The predominant genera were *Hydrogenophaga, Luteibacter*, and *Enhydrobacter* (Figure 1). After fractionation of this DNA extract in a CsCl gradient, generally the highest density from which a PCR product could be obtained was 1.74–1.75 g/mL, which corresponded to the expected peak of the ^13^C-labeled DNA in the SIP incubations. The community detected in this heaviest fraction of unincubated OSPW was much simpler, and dominated by the genera *Devosia, Methylomonas, Thauera*, and *Brevundimonas* (Figure 1). This OSPW-heavy community is a simplified subset of the OSPW-control community (Figure 1, Supplementary Table S1), suggesting that a few species have high G+C contents and therefore high DNA densities. This is supported by reported G+C contents for isolates of these genera: which range from 47 to 58% for *Methylomomas* (Hoefman et al., 2014), 64–6% for *Devosia* (Nakagawa et al., 1996), and 66–69% for *Thauera* (Mechichi et al., 2002).

Communities detected in the heavy DNA fractions of the SIP incubations were compared to two controls: the complete native OSPW community and the native community only in the 1.74–1.75 g/mL fraction, to verify that the species detected by SIP were truly enriched with ^13^C. Only OTUs that were enriched at least 10-fold in the SIP heavy fractions compared to both controls, or OTUs that were enriched at least 2-fold and were predominant at >10% of the total DNA in the heavy fraction, were considered to be enriched in the added ^13^C-substrate.

The ^13^C-labeled, heavy DNA fraction from the benzene-SIP experiment was dominated by a close relative of *Methyloversatilis universalis (Betaproteobacteria)* that accounted for 86.9% of the community and was enriched 20-fold compared to the OSPW-heavy control (Figure 1, Supplementary Table S1). *Zavarzinia (Alphaproteobacteria)* was the second most predominant genus, enriched 26-fold compared to OSPW-heavy controls. This result was reproducible: in a second SIP experiment of ^13^C-benzene incubated for 14 d *Methyloversatilis* was also predominant in the heavy DNA fraction (Supplementary Table S2). A SIP with ^13^C-methanol also verified the methylotrophic phenotype of this OTU (data not shown).

The three most dominant taxa in the ^13^C-naphthalene SIP heavy fraction were identified as *Chromatiaceae, Thauera*, and *Pseudomonas. Chromatiaceae* and *Pseudomonas* OTUs were enriched >25-fold compared to the OSPW-heavy control (Supplementary Table S1). The predominant *Thauera* OTU was enriched only 2-fold, but was the second most abundant OTU in this fraction. Although this *Thauera* OTU probably has a high G+C content and is therefore also abundant in the OSPW-heavy control, we conclude that its 2x enrichment in the benzene-heavy fraction may indicate a labeling of this organism by ^13^C for 2 reasons: (i) the absolute amount of DNA in the ^13^C-naphthalene SIP heavy fraction is much greater than the amount of DNA in the control OSPW-heavy fraction (31.6 vs. 0.17 ng/μl), indicating that this fraction is mostly newly synthesized DNA; and (ii) considering only OTUs that are many times enriched is a good approach for less common OTUs but may overlook common species, since abundances are relative and therefore the higher the initial abundance, the less it can increase (i.e., a greater than 10-fold increase is impossible for an OTU already present at 10% of the control, as seen for *Thauera*). Therefore the 2x enrichment of this OTU in the heavy fraction, combined with its predominance and the obvious increase in total DNA in the 1.74–1.75 fraction of the naphthalene-SIP, likely indicate that *Thauera* also metabolized the naphthalene.

### WIP-OSPW metagenome

**Figure 2:**
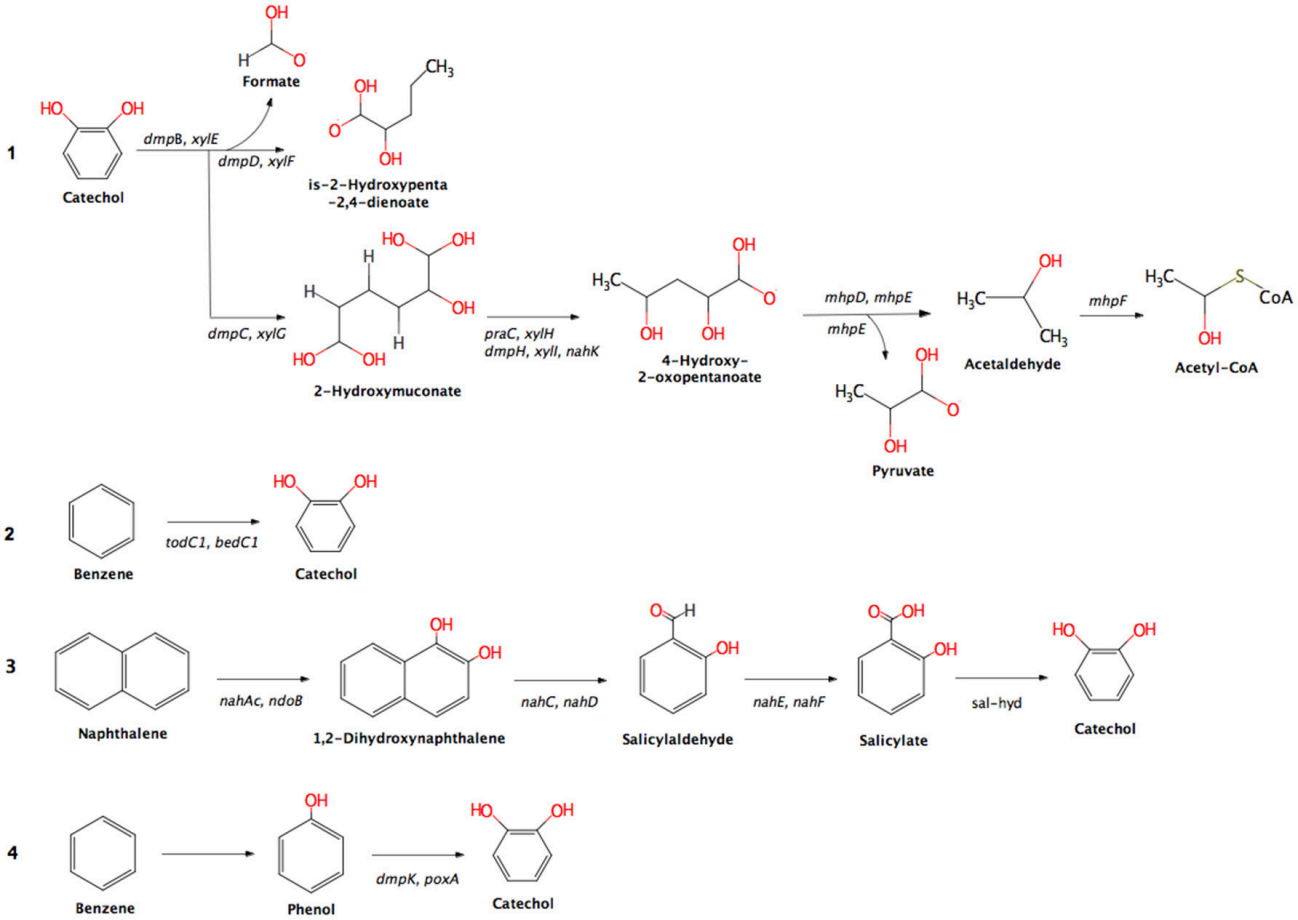
Detected pathways (substrates) for aerobic hydrocarbon metabolism in the OSPW metagenome and their associated genes. Pathway descriptions: 1, meta-cleavage of catechol; 2, dioxygenase and dehydrogenase reactions; 3, ring removal from polycyclic aromatic ring; and 4, two monooxygenase reactions. Enzyme names abbreviations and their synonyms: *dmpB, xylE*, catechol 2,3-dioxygenase; *dmpD, xylF*, hydroxymuconate-semialdehyde hydrolase; *dmpC, xylG*, aminomuconate-semialdehyde/2-hydroxymuconate-6-semialdehyde dehydrogenase; *praC, xylH*, oxalocrotonate tautomerase; *dmp*H, *xyl*I, *nah*K, 2-oxo-3-hexenedioate decarboxylase; *mhp*D, 2-keto-4-pentenoate hydratase; *mhp*E, 4-hydroxy 2-oxovalerate aldolase; *mhpF*, acetaldehyde dehydrogenase; *tod*C1, *bed*C1, benzene/toluene dioxygenase; *nahAc, ndoB*, naphthalene 1,2-dioxygenase; *nahC*, dihydroxynaphthalene dioxygenase; *nah*D, hydroxychromene-2-carboxylate isomerase; *nahE*, trans-o-hydroxybenzylidenepyruvate hydratase-aldolase; *nah*F, salicylaldehyde dehydrogenase; sal-hyd, salicylate hydroxylase; *dmpK, poxA*, phenol hydroxylase.

An overview of the OSPW metagenome is given in the Supplementary Table S3. A predicted 7.5% of the annotated genes belonged to the KEGG category “xenobiotics biodegradation and metabolism.” The metagenome shows the genetic machinery for aerobic degradation of diverse petroleum hydrocarbons. Aromatic and polyaromatic compounds are predicted to be broken down via three different aerobic pathways: dioxygenase and dehydrogenase reactions, ring removal from polycyclic aromatic ring structures, and two-monooxygenase reactions (Figure 2). Each process forms catechol as an intermediate, which is broken down through the catechol meta-cleavage pathway into Acetyl-CoA. Benzene is attacked in this scenario by either benzene/toluene dioxygenase, or by a monooxygenase then a phenol hydroxylase, while naphthalene is attacked by dihydroxynaphthalene dioxygenases (Figure 2).

**Figure 3:**
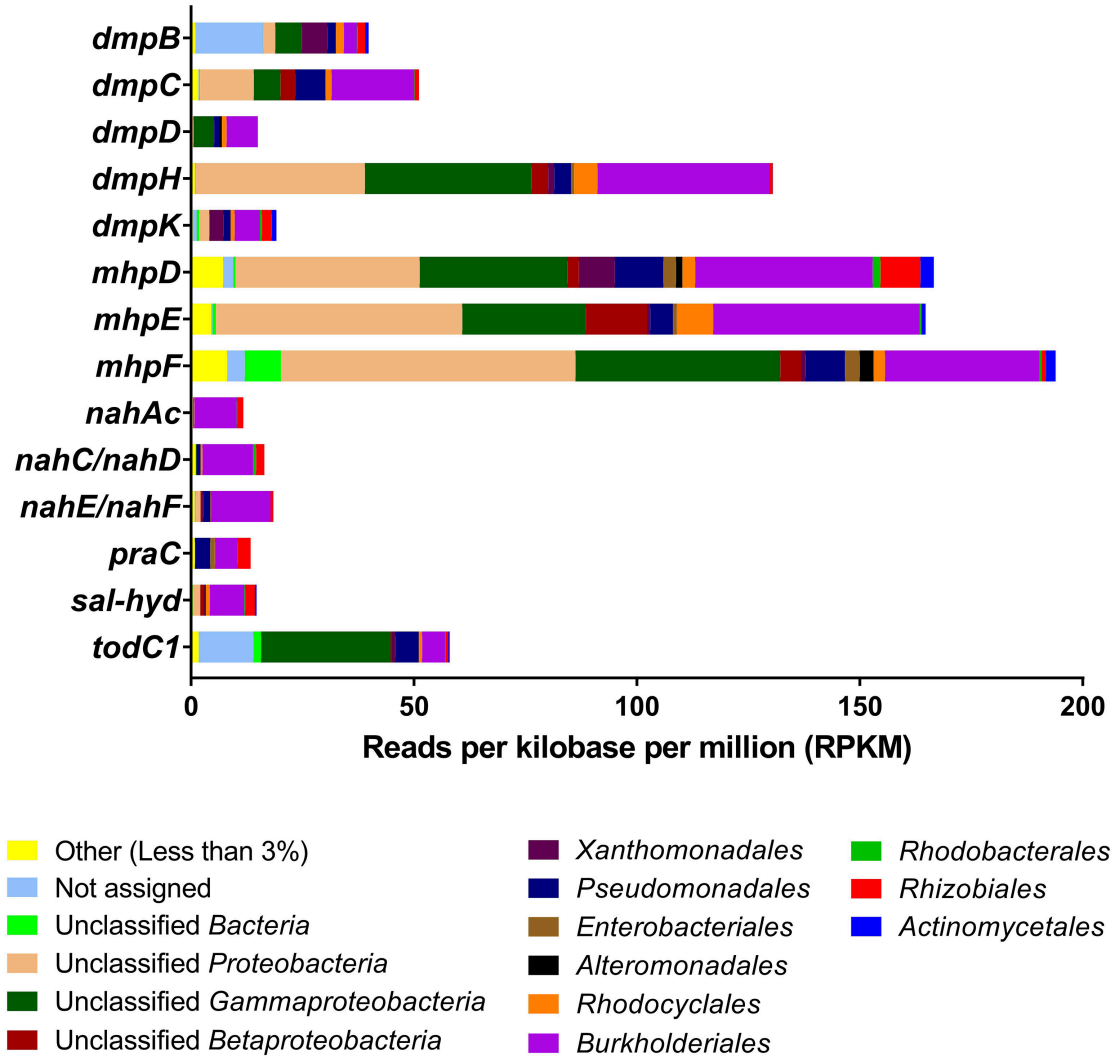
Unassembled metagenomic read distributions of the key genes from Figure 2. Bars represent reads per kilobase per million (RPKM) mapped to the corresponding gene using BLASTN. Taxonomic assignment on recruited reads that exceeded the 1% abundance cut-off was performed with MEGAN to the level of order. “Unclassified” groups include all members of a higher-level taxon that cannot be assigned at a more refined level. “Unassigned” represents reads MEGAN could not assign unambiguously to any taxon. RPKM values of genes *nahC* and *nahD*, and *nahE* and *nahF* are combined due to their identical functions and low RPKM levels.

**Figure 4:**
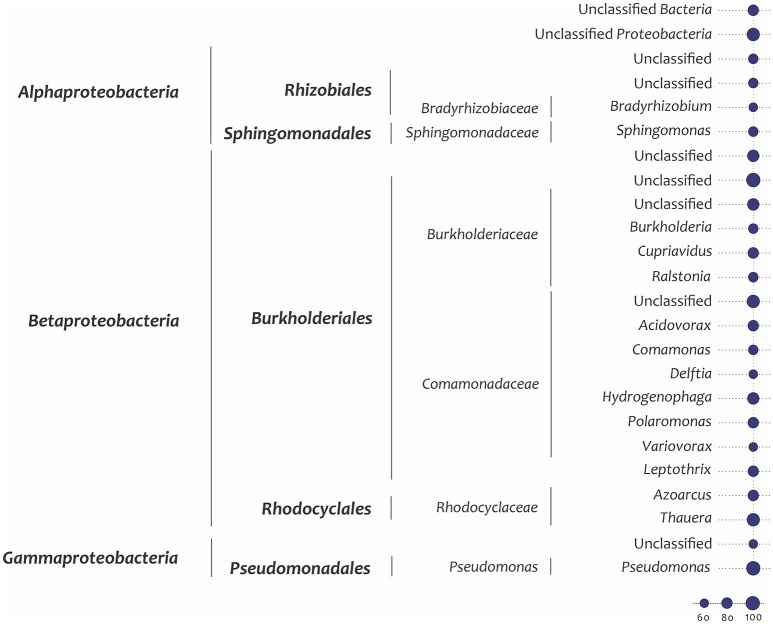
Prevalence of the 14 selected key marker genes encoding aromatic compound degradation within taxa. The prevalence parameter is calculated as the percentage of the gene sets in which a taxon is identified, and only taxa showing >60% prevalence (i.e.,>8 genes) are shown. “Unclassified” groups include all members of a higher-level taxon that cannot be assigned at a more refined level. The percent prevalence corresponds to the size of the node.

A quantitative analysis of unassembled Illumina metagenome reads for the key genes shown in Figure 2 suggested that these were predominantly found in *Betaproteobacteria* (particularly *Burkholderiales* and *Rhodocyclales*) and *Gammaproteobacteria* (particularly *Pseudomonadales* and unidentified orders) and a lesser extent in *Alphaproteobacteria and Actinobacteria* (Figure 3; finer taxonomic resolution of these results is shown for each gene in the Supplementary Figures S5A–P). The most unusual patterns were obtained for *dmp*B encoding for catechol 2,3-dioxygenase as well as *tod*C1 encoding benzene/toluene dioxygenase. Both showed a large number of hits to bacteria that could not be identified reliably, suggesting that at the enzyme level and possibly also the taxonomic level uncharacterized species are involved in the biodegradation of aromatics The unassembled Illumina read analysis was also used to calculate the most prevalent taxa within the set of key genes encoding aromatic-compound degradation (Figure 4). Prevalence of a taxon was determined as the proportion of the 14 raw read datasets (i.e., the 14 genes in Figure 2) a taxon was detected in. The taxa identified in Figure 4 are those with >60% of the key genes. They are shown to a higher taxonomic resolution than in Figure 3 because of the relative simplicity of the dataset. The most prevalent taxa were similar to the most abundant taxa, dominated by *Burkholderiales, Rhodocyclales, Pseudomonadales*.

Two of the bacterial taxa identified as important in the SIP studies, *Pseudomonas* (*Pseudomonadales*) and *Thauera (Rhodocyclales)*, show high abundance of some genes (Figure 3 and Supplementary Figure S5) and high prevalence of the overall gene set (Figure 4). The three other taxa identified as important in the SIP studies, *Methyloversatilis (Rhodocyclales), Chromatiaceae (Chromatiales)*, and *Zavarzinia (Rhodospirilalles)*, were neither abundant nor prevalent (Figures 3,4). However, in the case of *Methyloversatilis*, a large number of genes could be mapped to closely related sister genera within the *Rhodocyclales*/*Rhodocyclaceae* (*Azoarcus* and *Thauera*) (Figure 4; Supplementary Figures S5). MEGAN is limited by the NCBI database and is unable to resolve taxonomy unambiguously (Huson et al., 2007), so some of these sequences may in fact represent gene content of the important *Methyloversatilis* OTU. One member of the *Chromatiaceae* (*Chromatiales*) was detected as the closest BLAST hit to a catechol 2,3-dioxygenase and a benzene/toluene dioxygenase (data not shown).

### Isolated bacterial strains and their degradation potentials

Pure cultures of *Xanthobacter* (strain OSPW1) and *Zavarzinia* (strain OSPW2) were isolated using 20R2A-T medium (Supplementary Table S4). These were able to perform complete degradation of 6 μM benzene in 14 and 8 days, respectively, when growing on complex 20R2A medium (Supplementary Figure S6). *Thauera* sp. strain OSPW4 was isolated on complex media incubated under naphthalene vapor, which shows that it tolerates naphthalene, although it may not grow on naphthalene as a sole substrate. *Pseudomonas* sp. strain OSPW3 was isolated on mineral salts medium M10-T with naphthalene vapor as a sole substrate, and therefore was capable of growth on naphthalene as the sole substrate. No relatives of the two other predominant bacteria detected in ^13^C-benzene and ^13^C-naphthalene SIPs were isolated (i.e., *Methyloversatilis* and *Chromatiaceae*).

## Discussion

One plan for reclaiming oil sands tailings ponds in Canada is to convert them into End-Pit Lakes or other wetlands (Allen, 2008; Wayland et al., 2008; COSIA, 2016). This method uses a layer of freshwater in order to increase O_2_ supply (Allen, 2008; Wayland et al., 2008). It is expected that increased aeration will stimulate indigenous microbial decontamination rates, and that these systems will undergo natural biological remediation (Allen, 2008; COSIA, 2016). The capability of an OSPW community to aerobically degrade the model aromatic compounds benzene and naphthalene was demonstrated here. Naphthalene degradation rates were higher than those of benzene, which agrees with previous study showing that naphthalene has a higher biodegradation rate than other major hydrocarbons (Li and Goel, 2011). However, both rates are somewhat lower than previously measured rates in other oil-contaminated natural environments (Alvarez et al., 1991; Eriksson et al., 1999; Nicholson and Fathepure, 2005; Chang et al., 2014), probably because of the complexity of the hydrocarbon mixture in OSPW and the resultant low concentration of individual components. For example, the total PAH cocktail in this pond was previously estimated as 2600 ng L^−1^, but the naphthalene level only 101 ng L^−1^ (Madill et al., 1999). Although we used benzene and naphthalene as model compounds because of availability, these are only two components of a diverse mixture of aromatic compounds *in situ*.

SIP studies using ^13^C-benzene revealed the activity of the aerobic methylotroph genus *Methyloversatilis*. The genome of the type strain FAM5^T^ of *Methyloversatilis universalis* has a predicted membrane protein involved in aromatic hydrocarbon degradation through the metacleavage pathway (Kittichotirat et al., 2011). A recent study also discovered an uncultured bacterium related to *M. universalis* strain FAM5 (98% similarity) that can degrade benzene (Satija and Gore, 2014). Previous studies have also reported this bacterium in various petroleum related environments, but its relative abundance never reached more than 18% and its role in hydrocarbon degradation was not deemed crucial (Golby et al., 2012; Lenchi et al., 2013; Noguchi et al., 2014). Another prevalent genus identified in the heavy fractions of the ^13^C-benzene SIP experiment was related to *Zavarzinia* (Willems and De Vos, 2006). *Zavarzinia* is an aerobic carboxidotrophic bacterium (Meyer et al., 2015), and has also been detected in various hydrocarbon-contaminated sites (Nies and Schlegel, 1982; Al-Mailem et al., 2014, 2015). An isolate obtained in this study, named *Zavarzinia* sp. OSPW2, was shown to degrade benzene in pure culture (Supplementary Figure S7).

A different bacterial community was implicated in naphthalene degradation: predominantly *Gammaproteobacteria* of the family *Chromatiaceae, Gammaproteobacteria* of the genus *Pseudomonas*, and *Betaproteobacteria* of the genus *Thauera. Chromatiaceae* are abundant in OSPW surface water (2.6–8.0% relative abundance) (Saidi-Mehrabad et al., 2013). Some members of the family *Chromatiaceae* possess homogentisate 1,2-dioxygenase (HGD), a unique aromatic ring-cleavage enzyme, which cleaves an aromatic ring between *ortho* carbon atoms substituted with carboxyl and hydroxyl groups (Titus et al., 2000; Pérez-Pantoja et al., 2010). *Thauera phenylacetica* is a well-known facultative anaerobe that can degrade aromatic substrates under aerobic and denitrifying conditions (Mechichi et al., 2002). Similarly, many strains of *Pseudomonas stutzeri* have been studied biochemically and genetically for aerobic naphthalene degradation (Rosselló-Mora et al., 1994; Huang et al., 2015). Both a *Thauera* strain (OSPW4) and a *Pseudomonas* strain (OSPW3) were successfully cultivated from our WIP-OSPW sample supplemented with naphthalene, supporting the conclusions of the SIP study.

The use of metagenomics allowed us to identify genes encoding dioxygenases and monooxygenases for the aerobic degradation of aromatic and polyaromatic hydrocarbons in the OSPW community (Figures 2–4). At some level the metagenome data support the SIP experiments in identifying important bacterial groups. Five key taxa were identified as important in the SIP studies of benzene and naphthalene degradation: *Thauera, Pseudomonas, Methyloversatilis, Chromatiaceae* and *Zavarzinia. Three* of these: *Thauera, Pseudomonas*, and *Rhodocyclaceae* (which contains *Methyloversatilis*) were predominant in the analysis of metagenome genes encoding key enzymes of aromatic hydrocarbon degradation- both when assessing the number of reads mapping to these taxa and when assessing the percentage of genes in the gene set mapping to these taxa (Figure 3). That is, these taxa were predominant numerically, and contained most genes to encode the degradation pathways. A few genes related to catechol 2,3-dioxygenase, benzene/toluene dioxygenase, and phenol hydroxylase, and homogentisate 1,2-dioxygenase (HGD) in the metagenome were also mapped by BLAST to members of the *Chromatiaceae*. There are no genomes available for *Zavarzinia*, so the failure to identify genes belonging to it in the metagenome is unsurprising, although only a very few matches to its higher-level taxa occurred (*Rhodospiralles, Alphaproteobacteria*). This organism may be a rare member of the community that grew rapidly upon benzene amendment.

Therefore most of the key players identified in the SIP experiments appear to be well represented in the metagenome, although only *Thauera* and *Pseudomonas* would have been predicted from an analysis of the metagenome alone. An analysis of the metagenome independent of the SIP experiments would have proposed a large variety of other candidates as well, especially members of the *Burkholderiales*, which are very abundant in OSPW (An et al., 2013; Saidi-Mehrabad et al., 2013). In this study, *Burkholderiales* were predominantly associated with most of the genes for catechol meta-cleavage and other key functions of aromatics degradation found in the metagenome (Figure 3). Members of the *Rhizobiales* (*Alphaproteobacteria*) also appear to encode monooxygenases for degradation of aromatic compounds in the metagenome (Figures 3,4). Nevertheless, these two groups (*Burkholderiales* and *Rhizobiales*) did not appear as major active groups in either ^13^C-benzene or ^13^C-naphthalene SIPs. This finding indicates either that these genes cannot be properly mapped taxonomically with BLAST, or more likely that the detected genes are involved primarily in degradation of other abundant PAHs in the OSPW, rather than of the model aromatic compounds used in our experiments.

Therefore cultivation and SIP studies were key in filtering the huge mass of metagenomic data. Metagenomes alone have limited value for identifying bacterial species involved in particular processes, because: (i) mapping of key genes can usually only be inferred from BLAST identities, which is not always accurate due to incomplete pure-culture databases, as well as lateral gene transfer, (ii) some species may have the genetic systems required for a process but be only weakly active *in situ*, and (iii) a large number of genes detected (in our case ring-activating and ring-cleaving oxygenases) are not closely homologous to studied enzymes and their exact substrates are therefore uncertain. For example, benzene/toluene dioxygenase and phenol hydroxylase gene scaffolds in the metagenome were mapped to *Pseudomonas sp*. based on BLAST, but this bacterium was identified by SIP to be important in naphthalene but not benzene degradation. Naphthalene degradation genes from the metagenome were not mapped to *Chromatiaceae*, although this was identified by SIP as the most predominant organism in naphthalene degradation. There are clear limitations of the homology-based metagenome analysis.

The coupling of metagenomic sequencing technology, SIP, and traditional microbiological methods allowed us to identify organisms with important roles in degrading model aromatic compounds within the OSPW. Utilizing the metagenome alone was not very useful, since it predicted a huge number of potential hydrocarbon degraders (mostly *Burkholderiales* and *Rhizobiales*), but only a few of these were shown to be active in the actual SIP experiments using ^13^C-benzene and ^13^C-naphthalene. The others presumably utilize other substrates or are active under other conditions. Some predominant bacteria found to be important in our benzene and naphthalene amended environments, such as *Methyloversatilis* and *Chromatiaceae*, were not predominant in other related studies (Pérez-Pantoja et al., 2010; Patel et al., 2012; Jechalke et al., 2013; Ortega-González et al., 2013; Hassanshahian and Boroujeni, 2016), demonstrating the uniqueness of this environment.

## Author contributions

FR, IT, JK, LG, and PD designed the experiments. FR conducted the majority of the experimental work including SIP, DNA processing, and biochemical work, assisted by IT, JK, LG and ASM. Bioinformatics analysis were performed by AS, IT, XD, and CS. FR and AS designed the figures. FR and PD wrote the manuscript. All authors discussed the findings and provided input on the final manuscript.

### Conflict of interest statement

The authors declare that the research was conducted in the absence of any commercial or financial relationships that could be construed as a potential conflict of interest.
