# Supplementary material for: Benzene and Naphthalene Degrading Bacterial Communities in an Oil Sands Tailings Pond
Source: Front Microbiol. 2017 Sep 28;8:1845. doi: 10.3389/fmicb.2017.01845 (PMC5627004; doi:10.3389/fmicb.2017.01845)
Supplement: Supplementary file 1 [file Presentation1.pdf]

Supplementary Information File

Benzene and naphthalene degrading bacterial communities in an oil sands tailings pond

Fauziah F Rochman[1], Andriy Sheremet[1], Ivica Tamas[1,2], Alireza Saidi-Mehrabad[1,3], Joong-Jae Kim[1], Xiaoli Dong[4,5], Christoph W Sensen[4,6], Lisa M Gieg[1], Peter F Dunfield[1]*

[1]Department of Biological Sciences, University of Calgary, 2500 University Drive NW, Calgary, Alberta, Canada, T2N 1N4
[2]Department of Biology and Ecology, Faculty of Sciences, University of Novi Sad, Trg Dositeja Obradovića 2, 21000 Novi Sad, Serbia.
[3]Department of Biological Sciences, University of Alberta, Edmonton, Alberta, T6G 2E1, Canada
[4]Department of Biochemistry & Molecular Biology in the Cumming School of Medicine, University of Calgary, Calgary, Alberta, T2N 1N4, Canada
[5]Department of Geoscience, University of Calgary, 2500 University Drive NW, Calgary, Alberta, Canada, T2N 1N4
[6]Institute of Computational Biotechnology, Graz University of Technology, Petersgasse 14, 8010 Graz, Austria

Correspondence: PF Dunfield Tel: 403-220-2469 Fax: 403-289-9311 E-mail: pfdunfie@ucalgary.ca

Supplementary Figure S1 Density gradients of DNA extracts from SIP experiments using WIP-OSPW sampled in August, 2011, with and without the addition of ^{13}C-labeled model hydrocarbons: benzene after 9 d (A) and naphthalene after 7 d (B). Twelve density fractions are shown as filled circles (amended with substrates) and empty circles (DNA from OSPW tailings water or control). Fractions indicated by arrows were subject to microbial community analyses. Black arrows indicate the control fraction 5 (OSPW-heavy) used in this study.

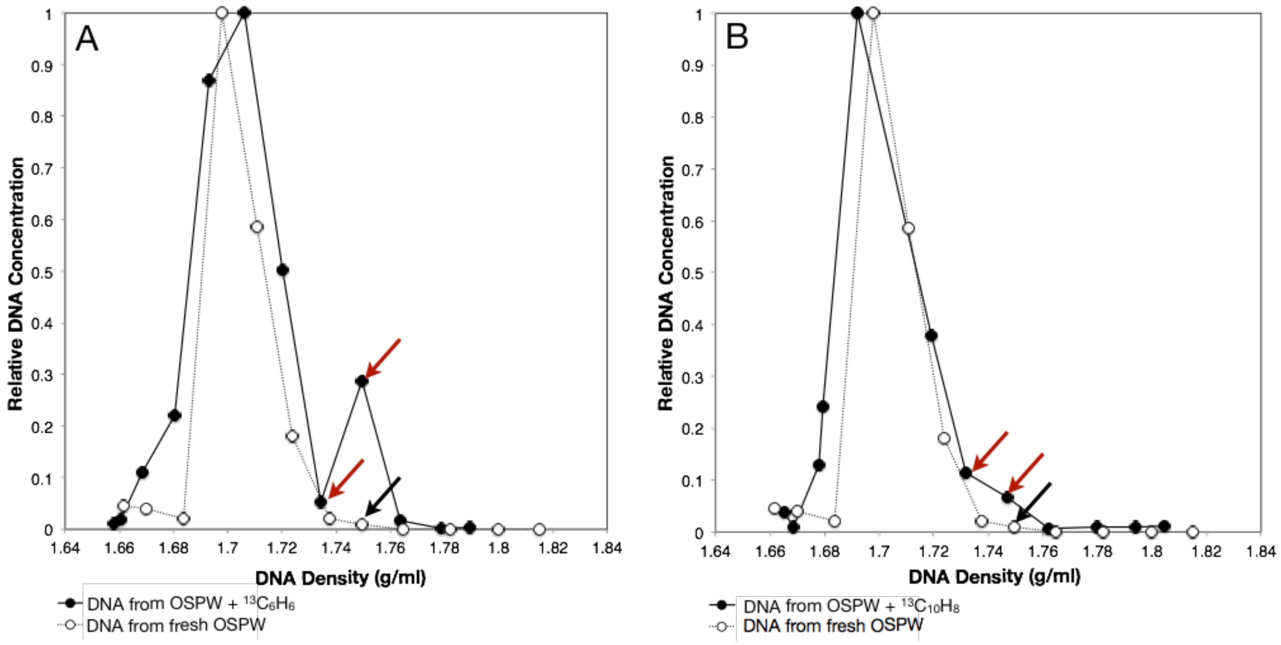

Supplementary Figure S2 – A Potential benzene oxidation of OSPW sampled in August 2011, with the results of linear regressions for benzene oxidation. n = 3, bars = standard error of the mean, bars smaller than the symbols are not visible. The results of linear regression are shown at the bottom of the graph. The regression converts to a total benzene oxidation rate of 4.3 μmol L^{-1} OSPW d^{-1}, using Henry's law to calculate the amount of benzene in the liquid (20 ml) and gas (90 ml) phases (See Methods).

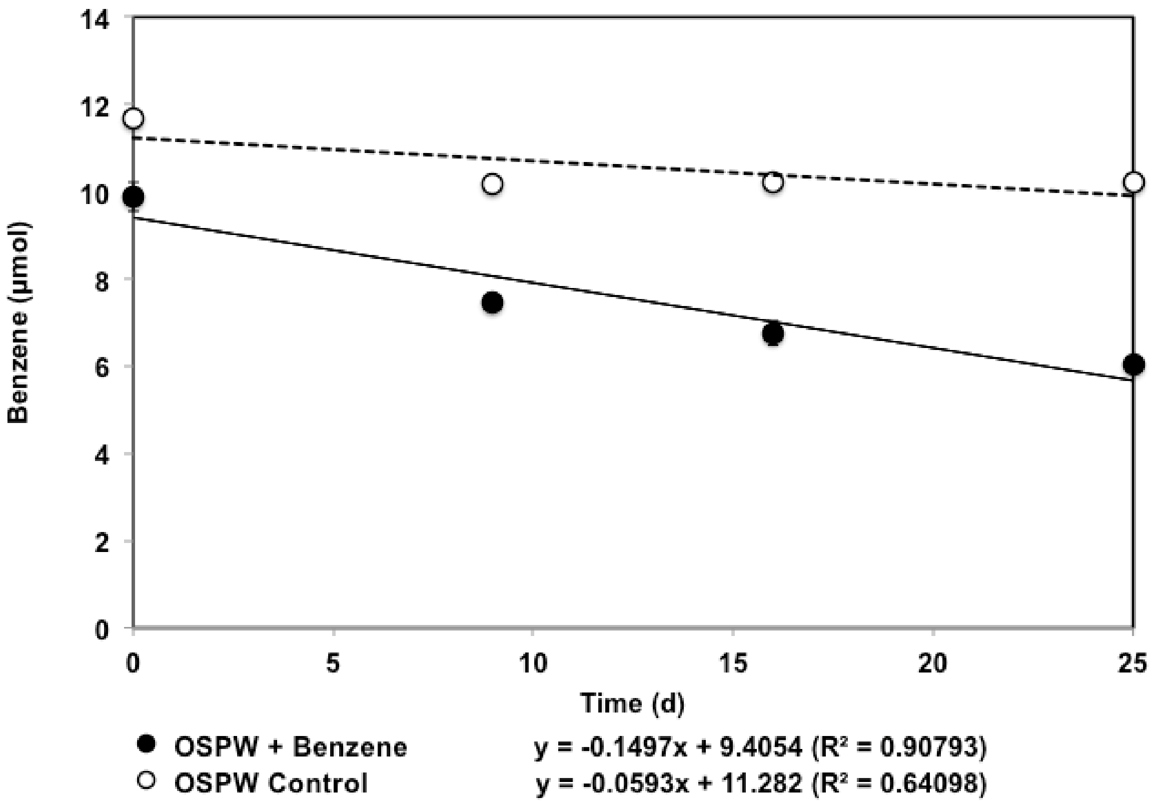

Supplementary Figure S2 – B CO_2 production time courses of triplicate samples from OSPW amended with benzene, sampled in August 2011. n = 3, bars = standard error of the mean, bars smaller than the symbols are not visible. The regression converts to a total CO_2 production rate of 5.6 μmol L^{-1} OSPW d^{-1}, using Ostwald Coefficient to calculate the amount of CO_2 in the liquid (20 ml) and gas (90 ml) phases.

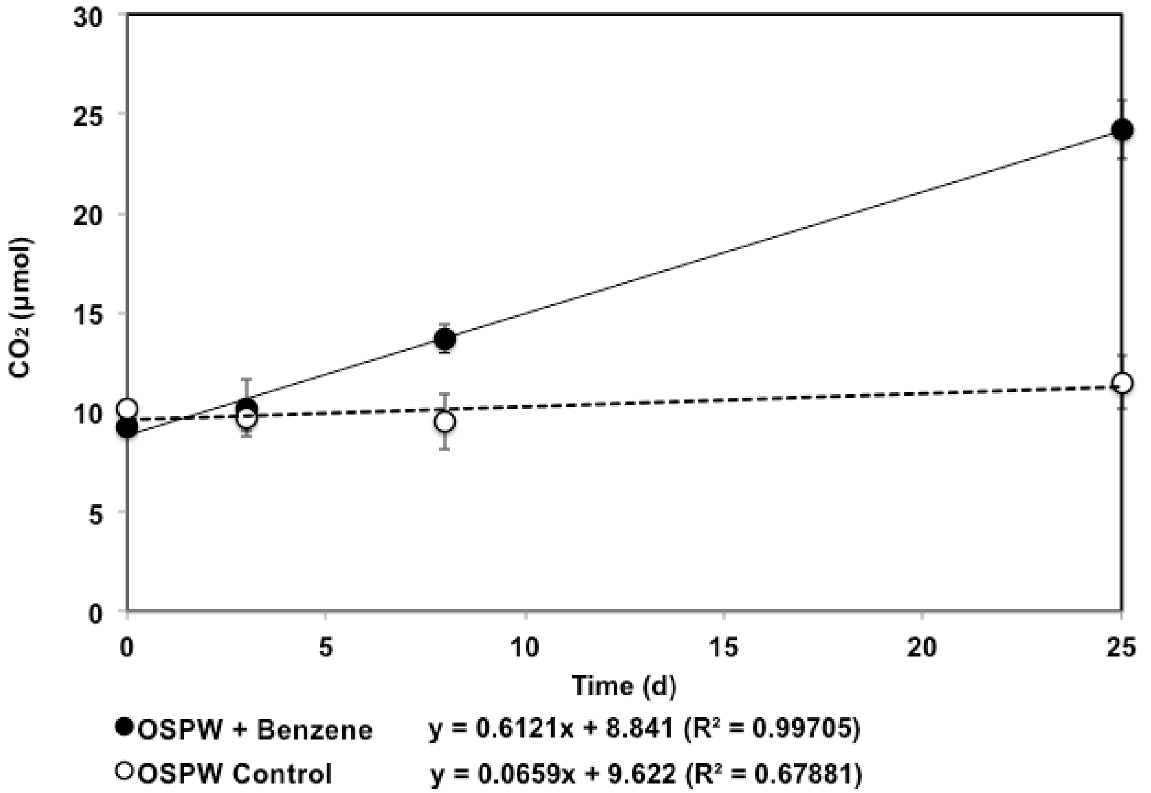

Supplementary Figure S3 A Naphthalene oxidation of OSPW sampled in August, 2011, with the results of linear regressions. The y-axis represents the total amount of naphthalene localized in the 2, 2, 4, 4, 6, 8, 8-heptamethylnonane carrier. n = 3, bars = standard error of the mean, bars smaller than the symbols are not visible. The regression converts to a total naphthalene oxidation rate of 21.4 μmol L^{-1} OSPW d^{-1} in the 40-mL OSPW samples.

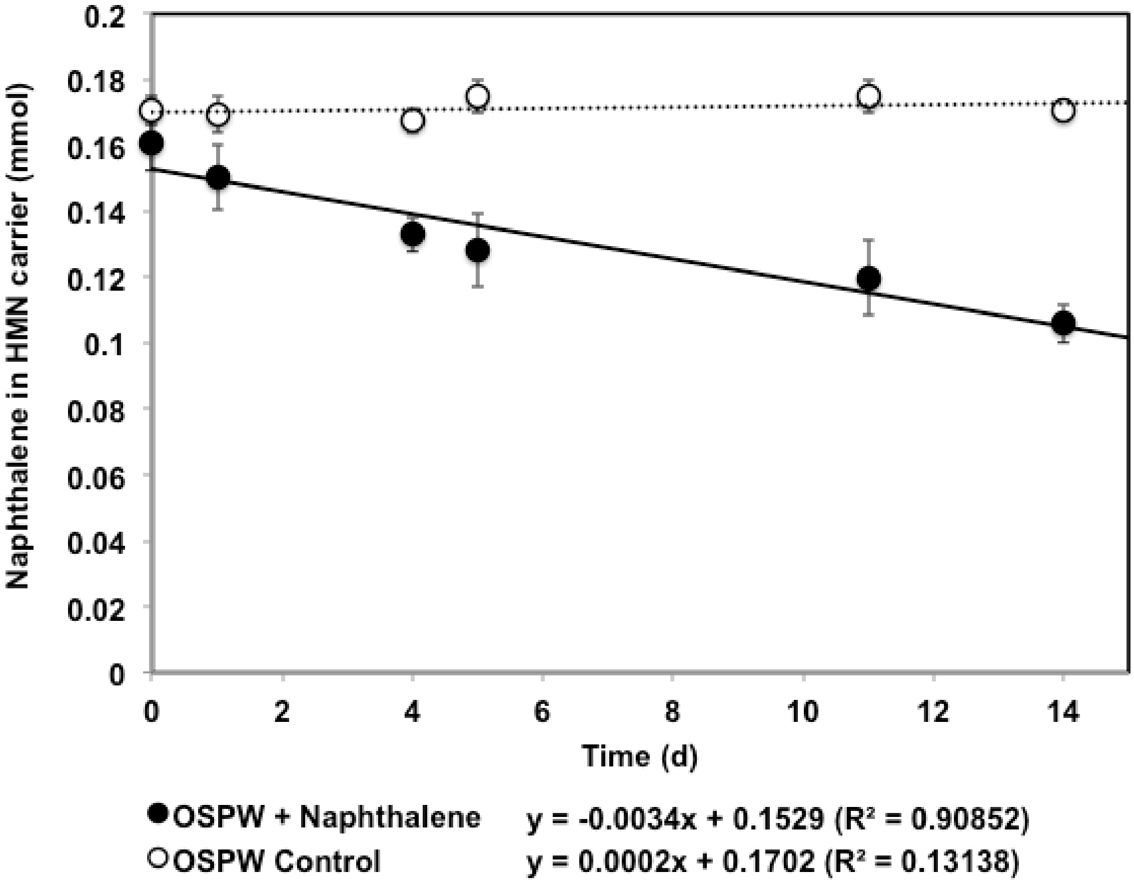

Supplementary Figure S3 B CO_2 production time courses of triplicate samples from OSPW amended with naphthalene, sampled in August 2011. n = 3, bars = standard error of the mean, bars smaller than the symbols are not visible. The regression converts to a total CO_2 production rate of 144.1 μmol L^{-1} OSPW d^{-1}, using Ostwald Coefficient to calculate the amount of CO_2 in the liquid (20 ml) and gas (90 ml) phases.

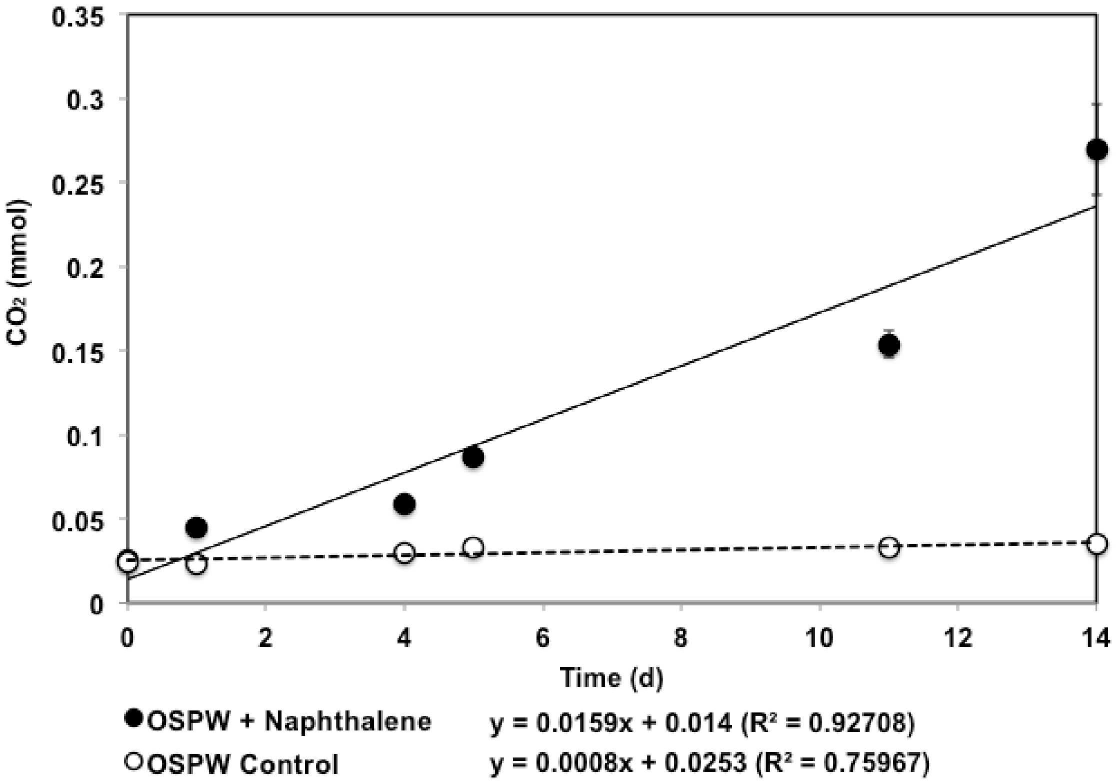

Supplementary Figure S4 Predominant classee detected in DNA of control OSPW samples, and in heavy DNA fractions extracted after incubation with ^{13}C benzene or ^{13}C naphthalene. Data are relative abundances of taxa within sequenced 16S rRNA gene amplicons (only classes >1% of the total reads are shown). Benzene: heavy-DNA fraction of benzene-amended OSPW incubated for 9 d; Naphthalene: heavy-DNA fraction of naphthalene- amended OSPW incubated for 7 d; OSPW-heavy: heavy fraction of OSPW incubated for the same amount of time as the amended samples; and OSPW-control: complete, unfractionated DNA from OSPW. The bubbles show 6 abundance classes (1-1.75%; 1.76-4.5%; 4.6-9%; 9.1-18.5%; 18.6-37.5%; >37.6%).

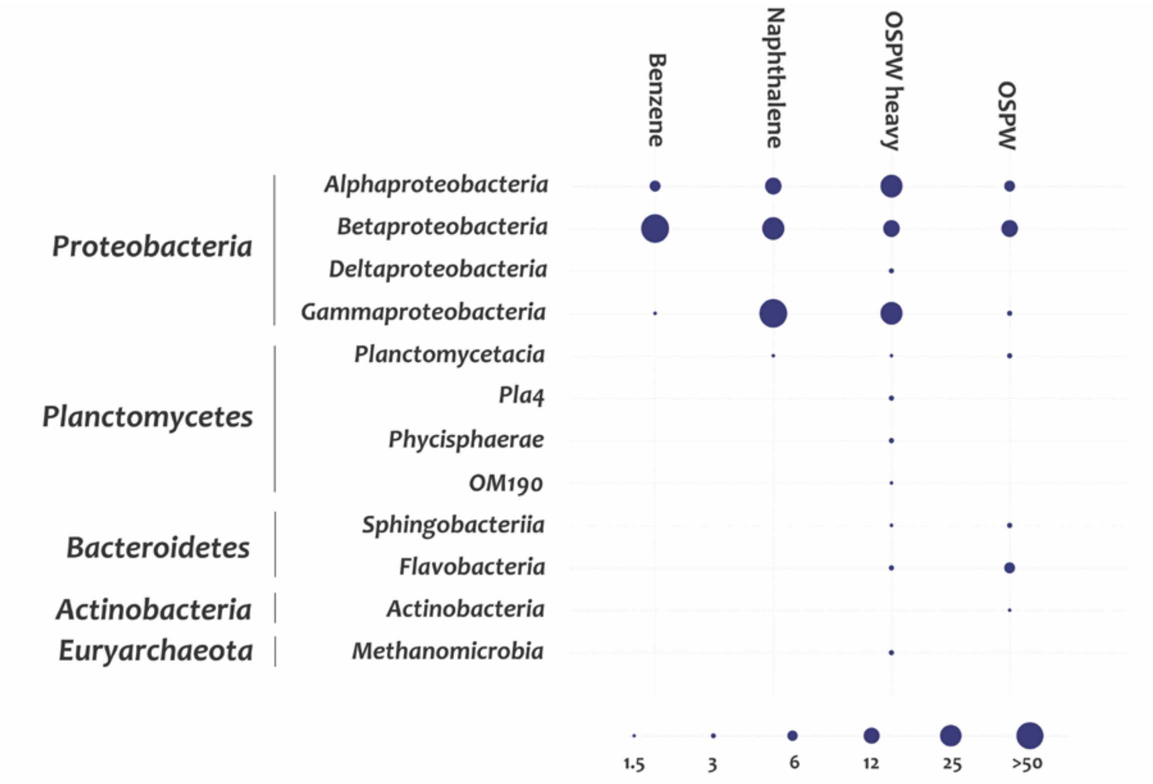

Supplementary Figure S5 A – P Taxonomic assignments to the level of family, based on best BLAST hits of the metagenomic reads mapped to selected genes encoding enzymes for aerobic benzene/naphthalene degradation. Read recruitment was performed using BLASTN; the MEGAN software was used for taxonomy assignment. Numbers indicate the number of reads assigned to a taxon at a given level, and correspond to the node sizes displayed on the trees. Nodes are not cumulative, e.g. the *Proteobacteria* node only includes reads not identified at a higher taxonomic resolution.

Supplementary Figure S5 A *dmpB* (catechol 2,3-dioxygenase).

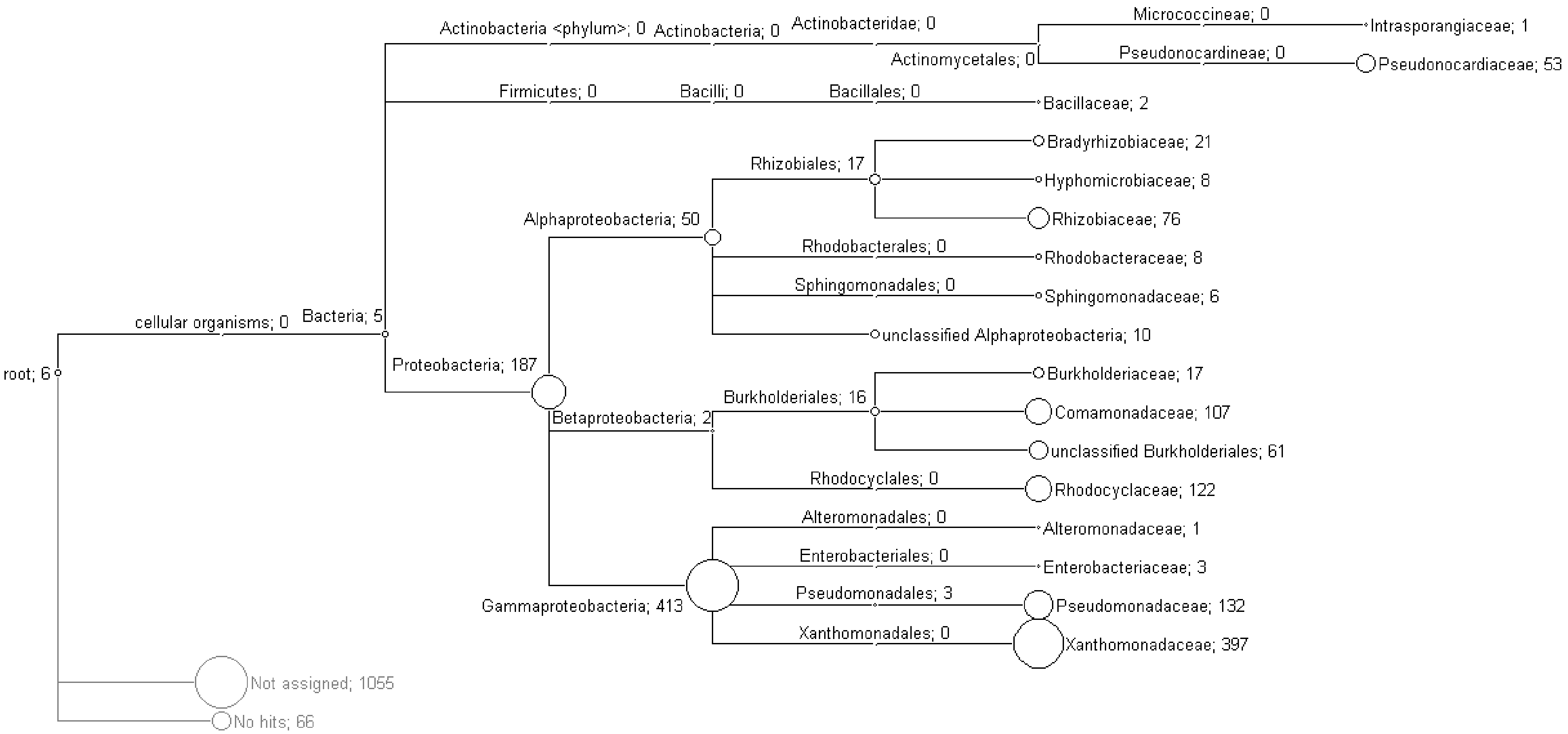

Supplementary Figure S5 B *dmpC* (aminomuconate-semialdehyde/2-hydroxymuconate-6-semialdehyde dehydrogenase).

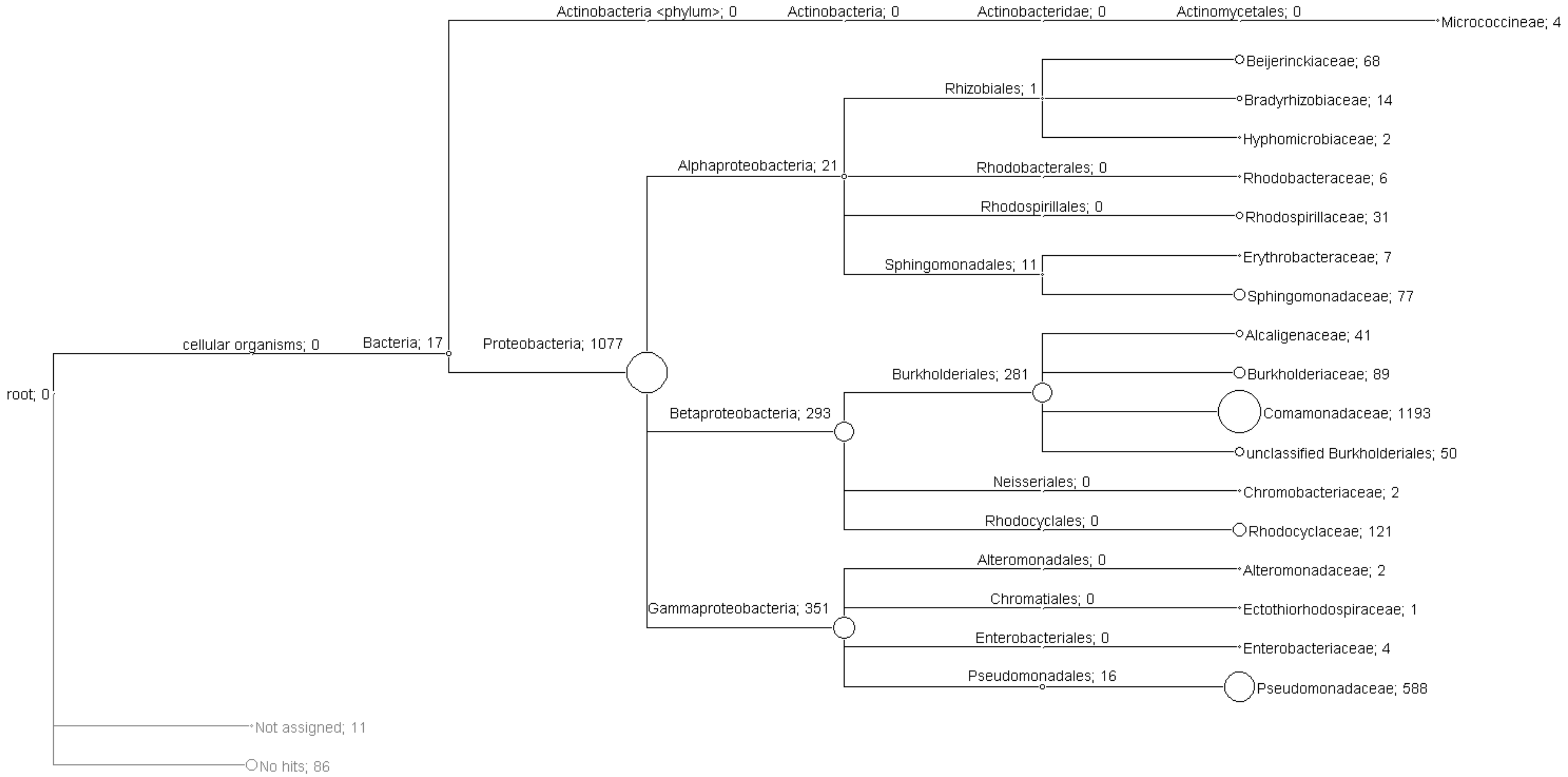

Supplementary Figure S5 C *dmpD* (hydroxymuconate-semialdehyde hydrolase).

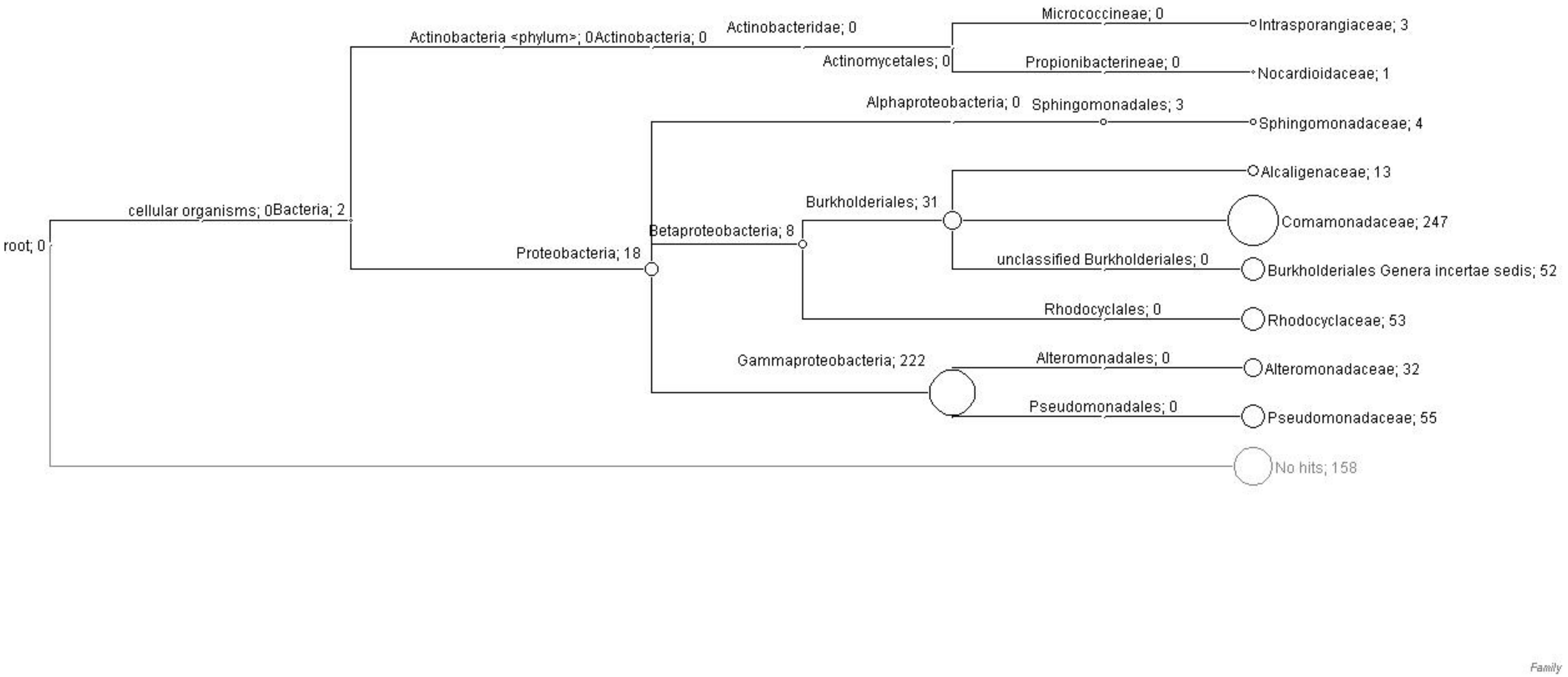

Supplementary Figure S5 D *dmpH* (2-oxo-3-hexenedioate decarboxylase).

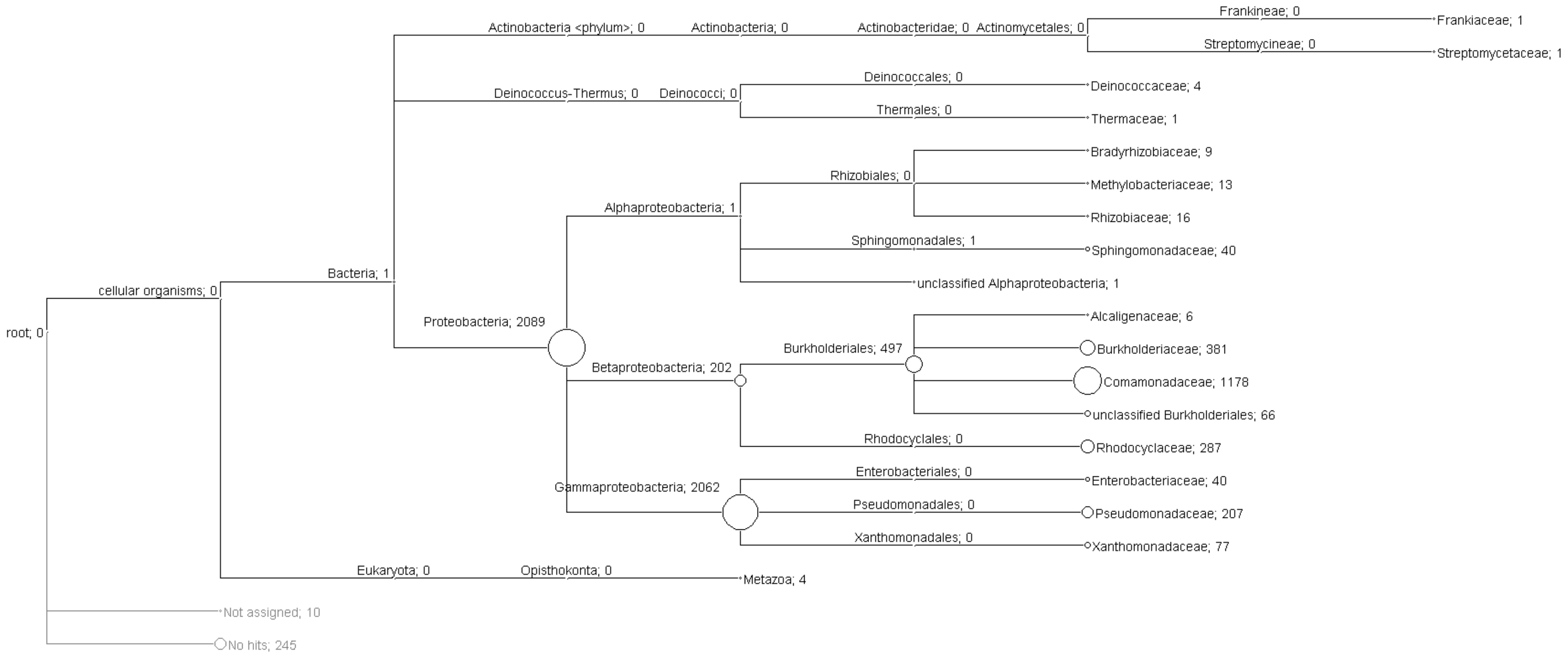

Supplementary Figure S5 E *dmpK* (phenol hydroxylase).

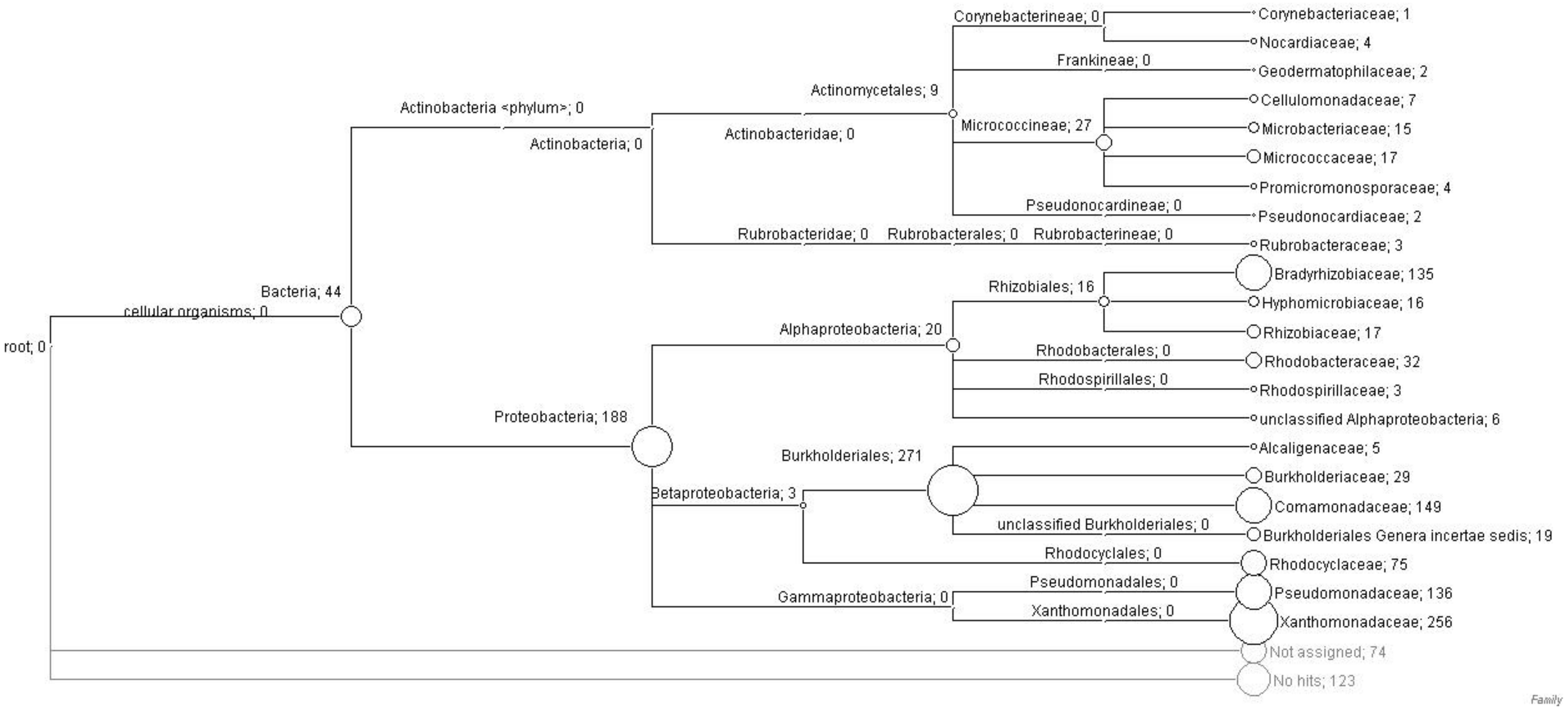

Supplementary Figure S5 F *mhpD* (2-keto-4-pentenoate hydratase).

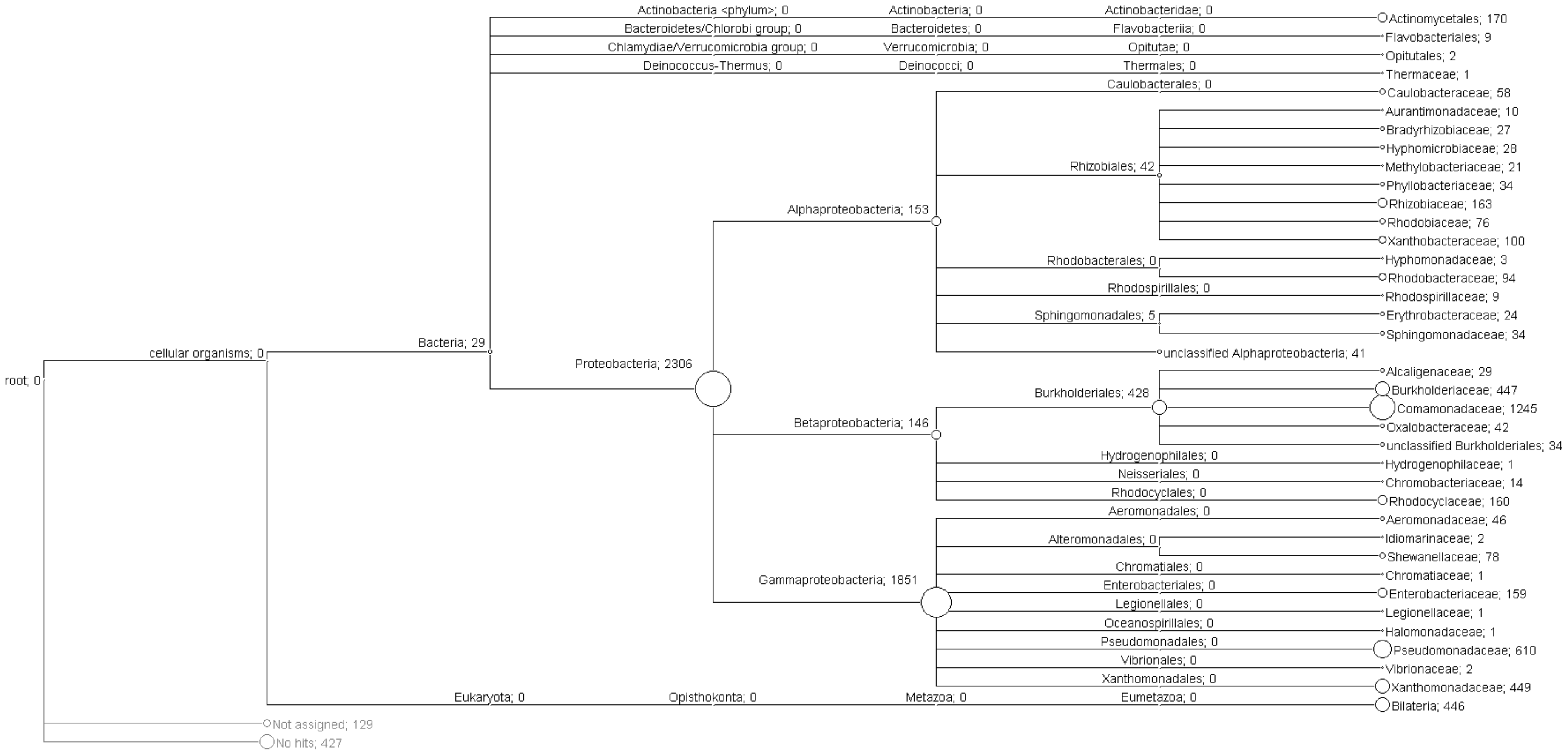

Supplementary Figure S5 G *mhpE* (4-hydroxy 2-oxovalerate aldolase).

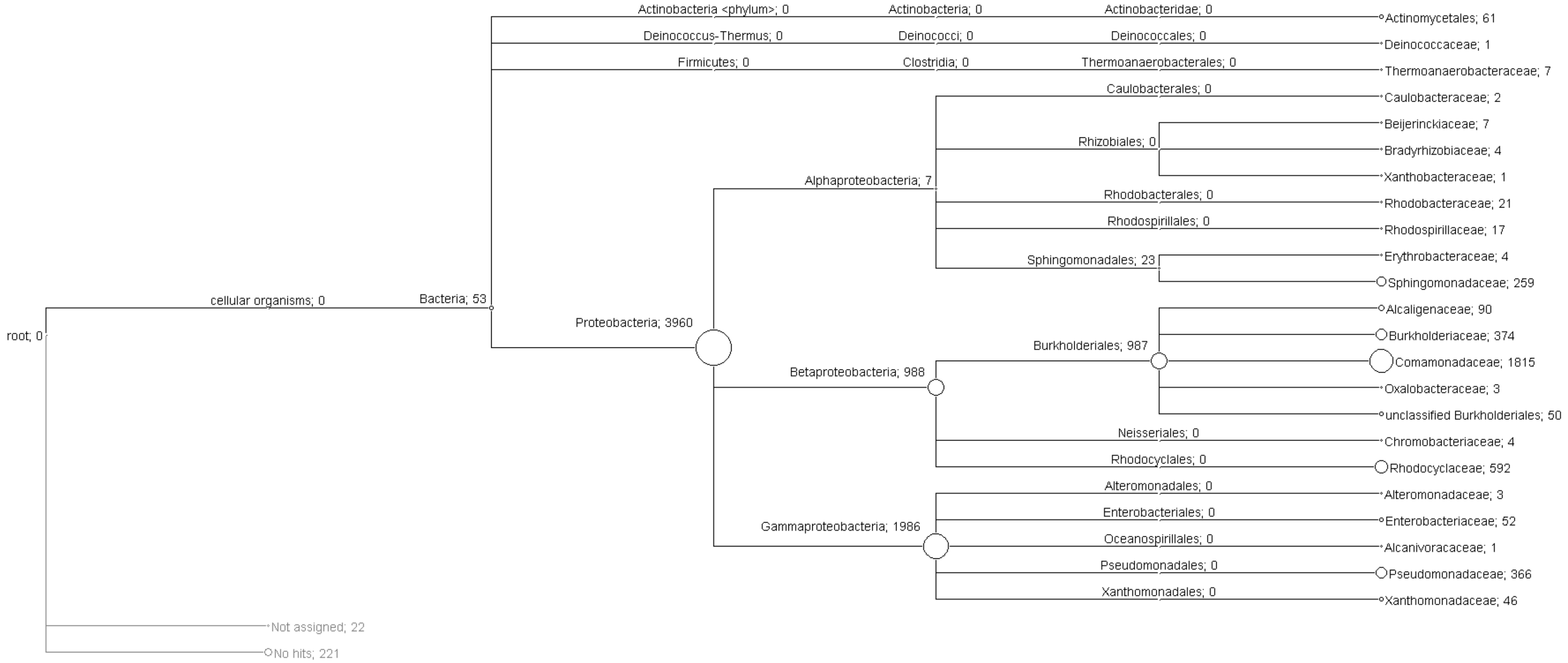

Supplementary Figure S5 H *mhpF* (acetaldehyde dehydrogenase).

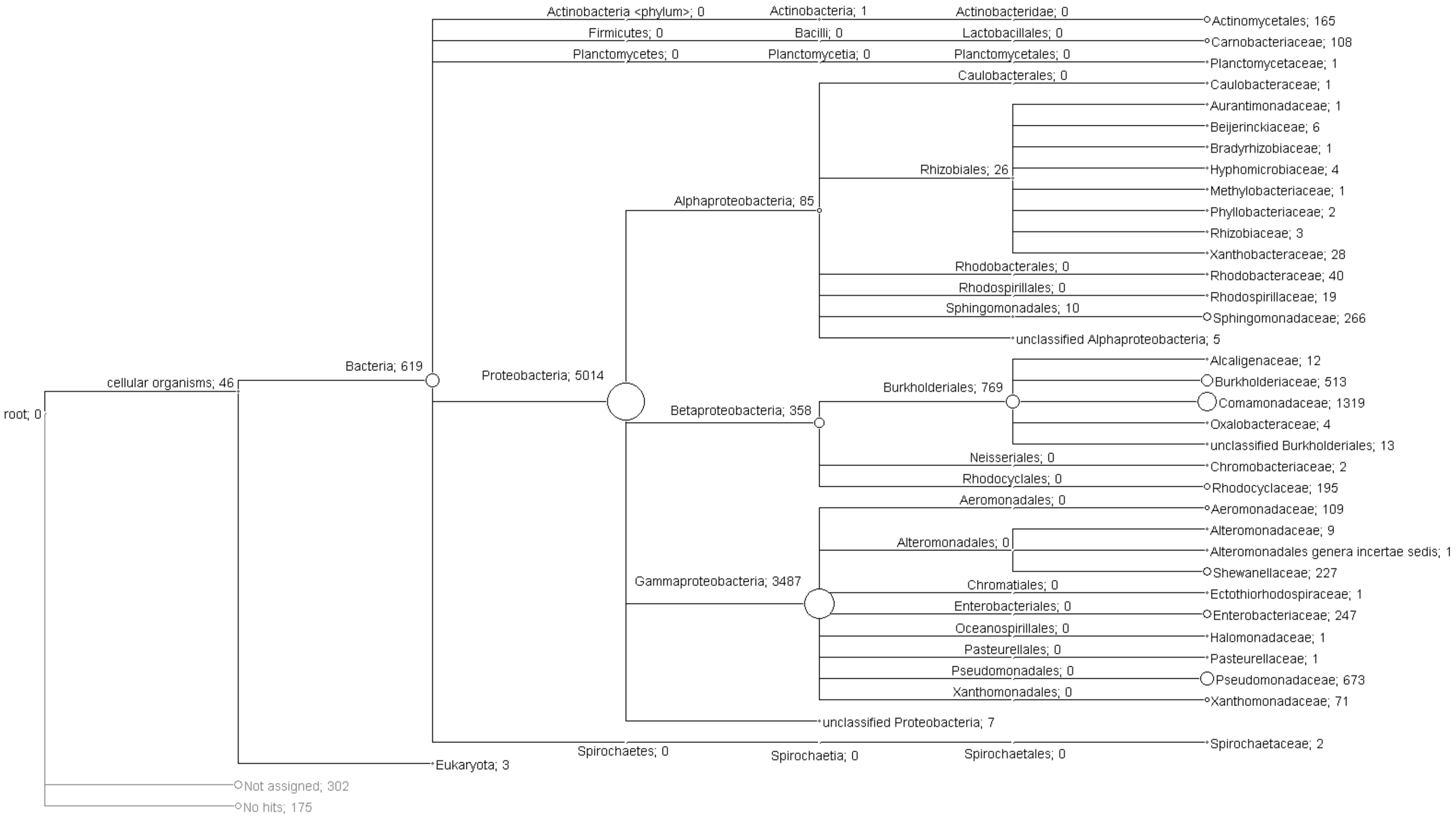

Supplementary Figure S5 I *nahAc* (naphthalene 1,2-dioxygenase).

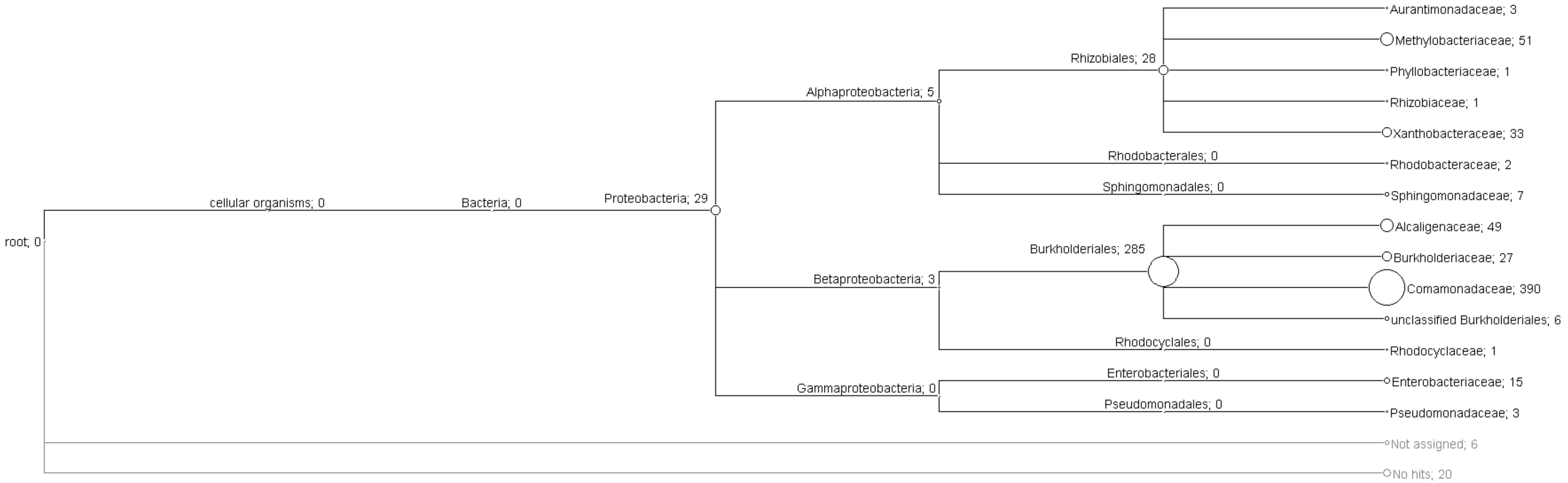

Supplementary Figure S5 J *nahC* (dihydroxynaphthalene dioxygenase).

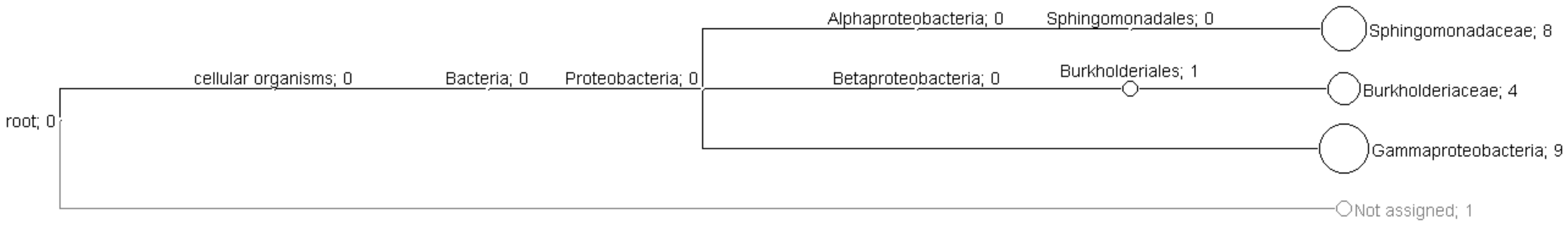

Supplementary Figure S5 K *nahD* (hydroxychromene-2-carboxylate isomerase).

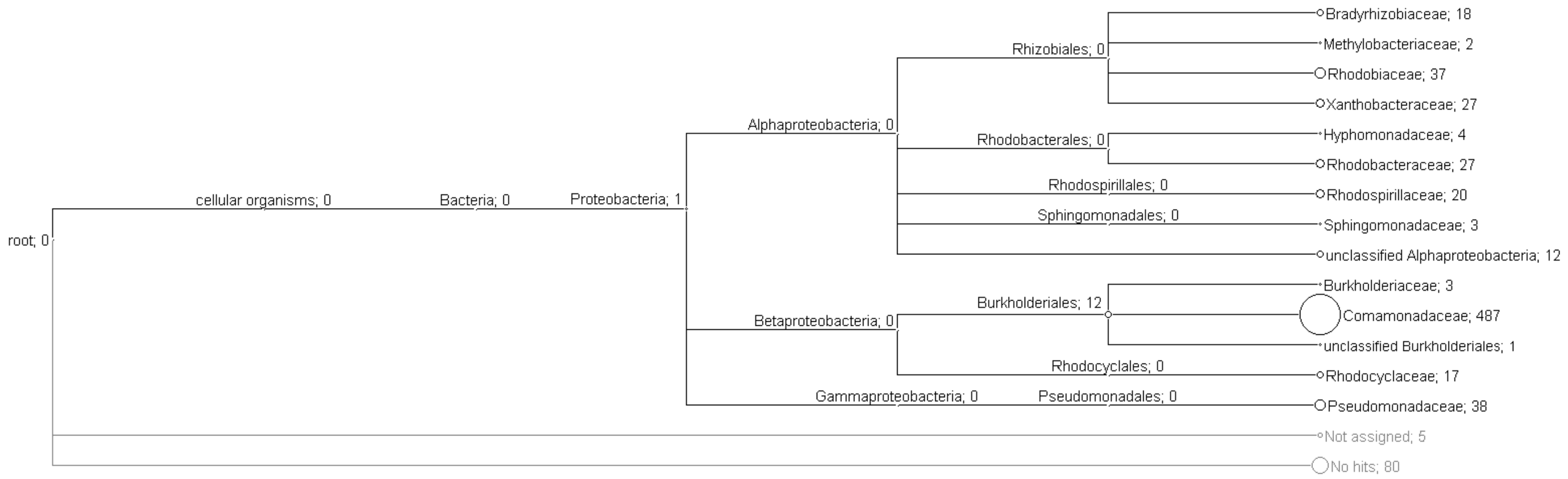

Supplementary Figure S5 L *nahE* (trans-o-hydroxybenzylidenepyruvate hydratase-aldolase).

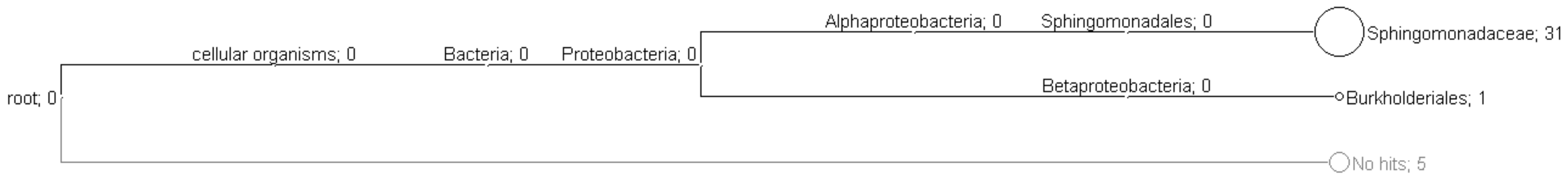

Supplementary Figure S5 M *nahF* (salicylaldehyde dehydrogenase).

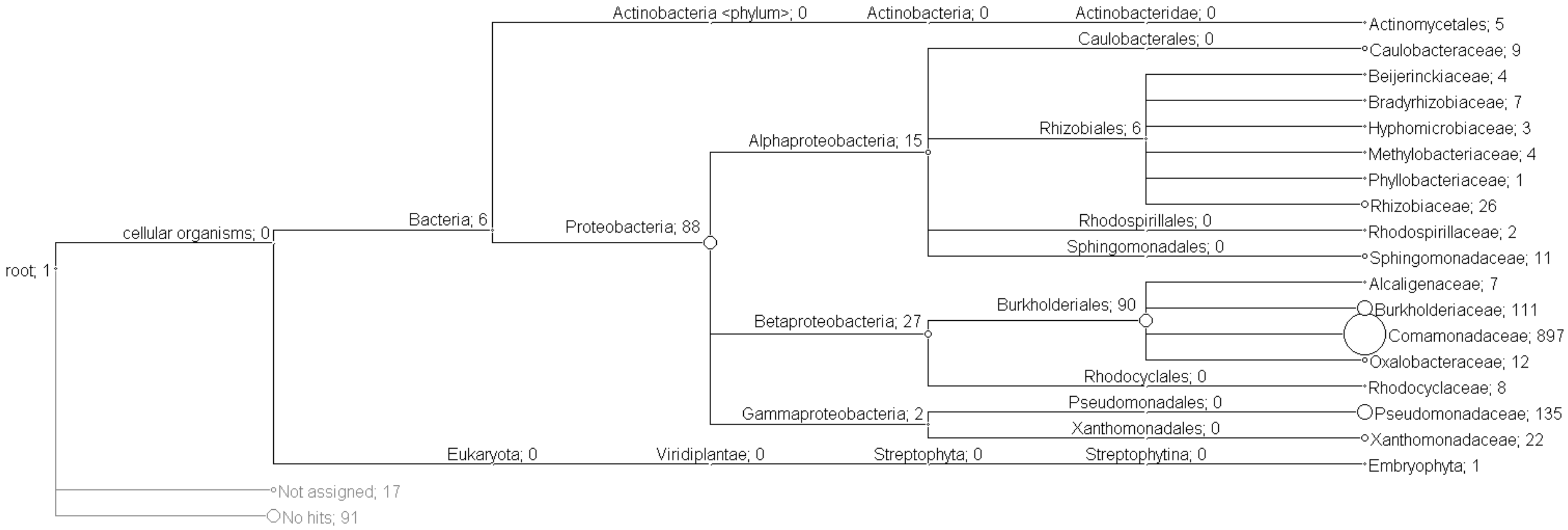

Supplementary Figure S5 N *praC* (oxalocrotonate tautomerase).

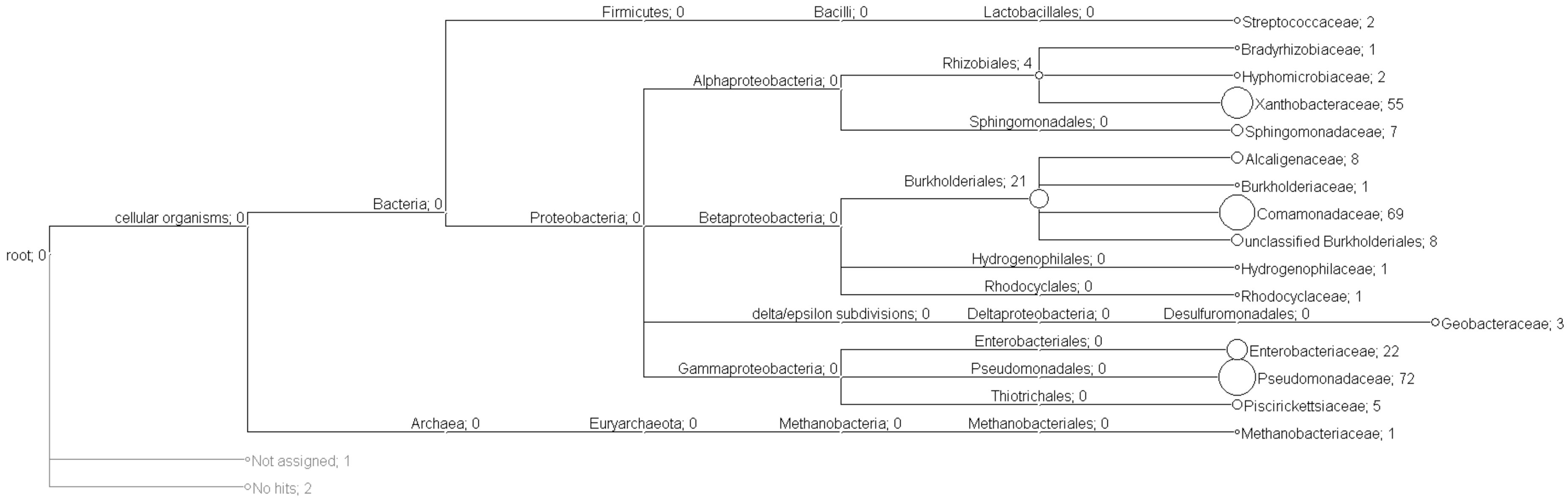

74 **Supplementary Figure S5 O** *sal-hyd* (salicylate hydroxylase).

75

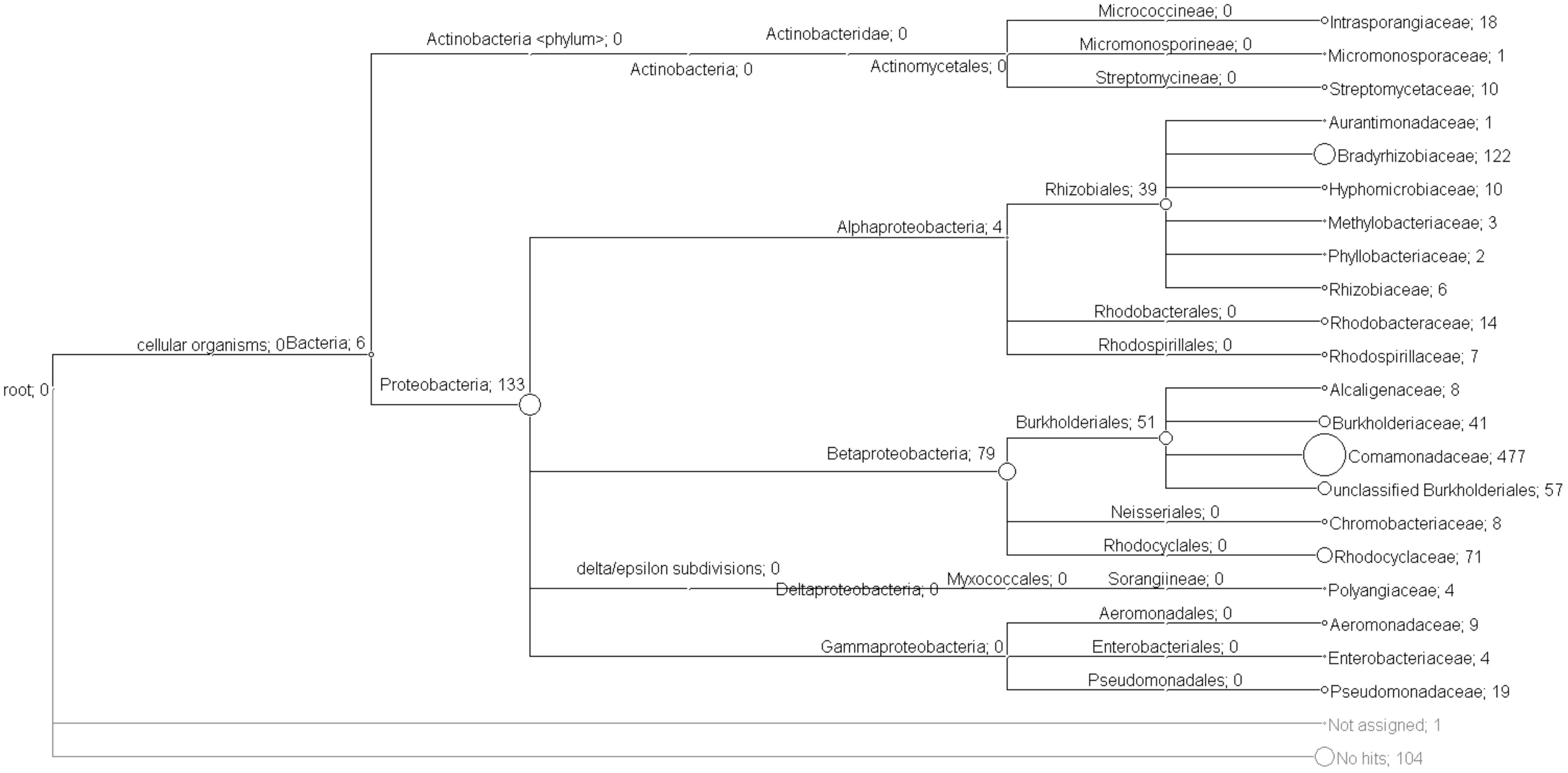

76

Supplementary Figure S5 P *todC1* (benzene/toluene dioxygenase).

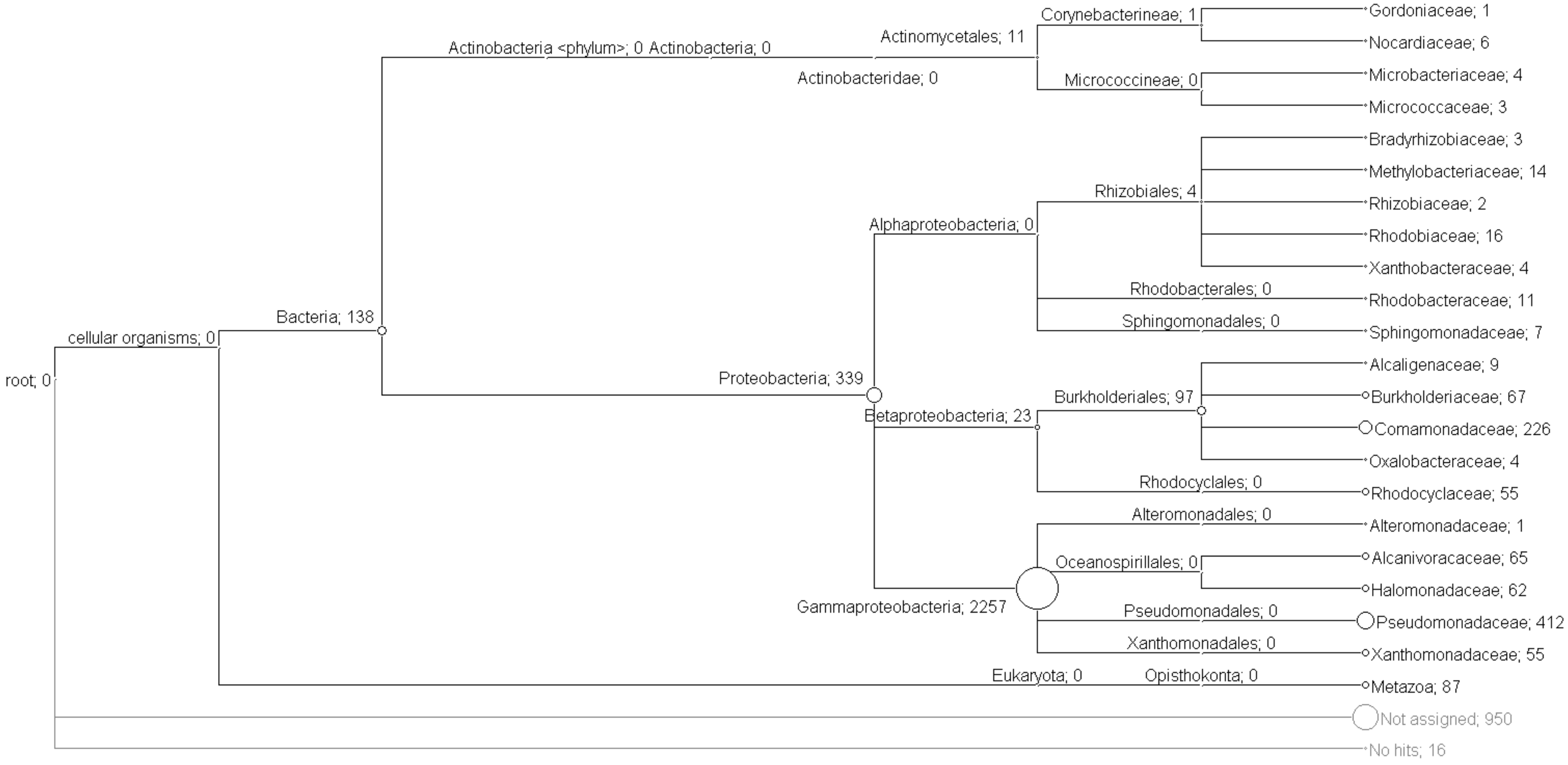

Supplementary Figure S6 Benzene degradation by pure cultures of *Xanthobacter* sp. OSPW1 and *Zavarzinia* sp. OSPW2 isolated from OSPW. Experiments were done in triplicates. Data are means ± standard errors of the mean.

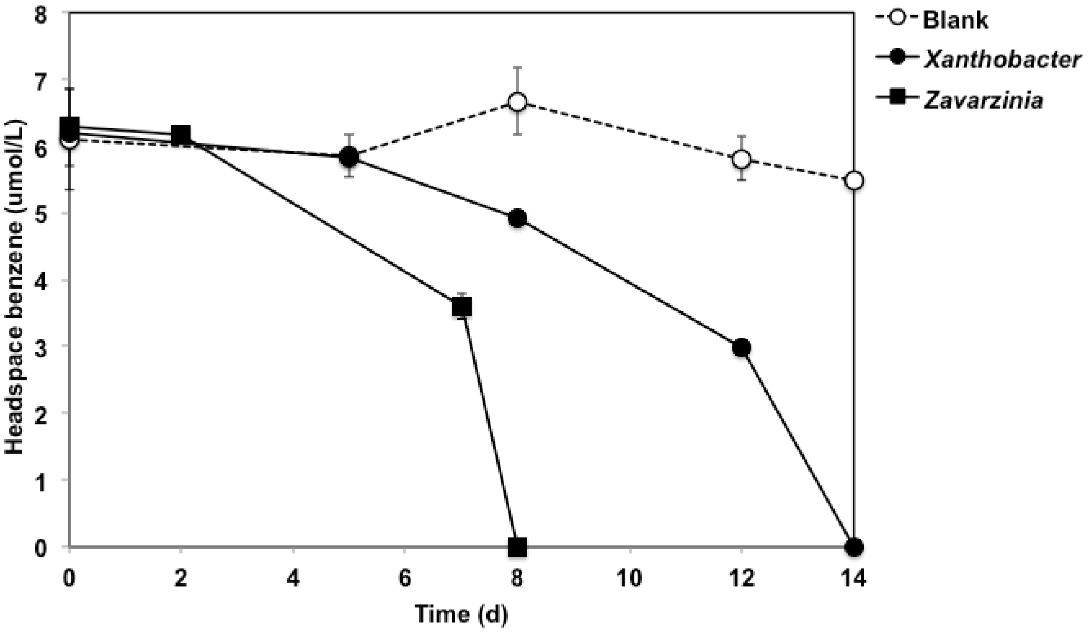

Supplementary Figure S7 Comparison between read counts for the 14 marker genes obtained for Illumina and 454 sequencing platforms. Each point on the graph corresponds to a gene.

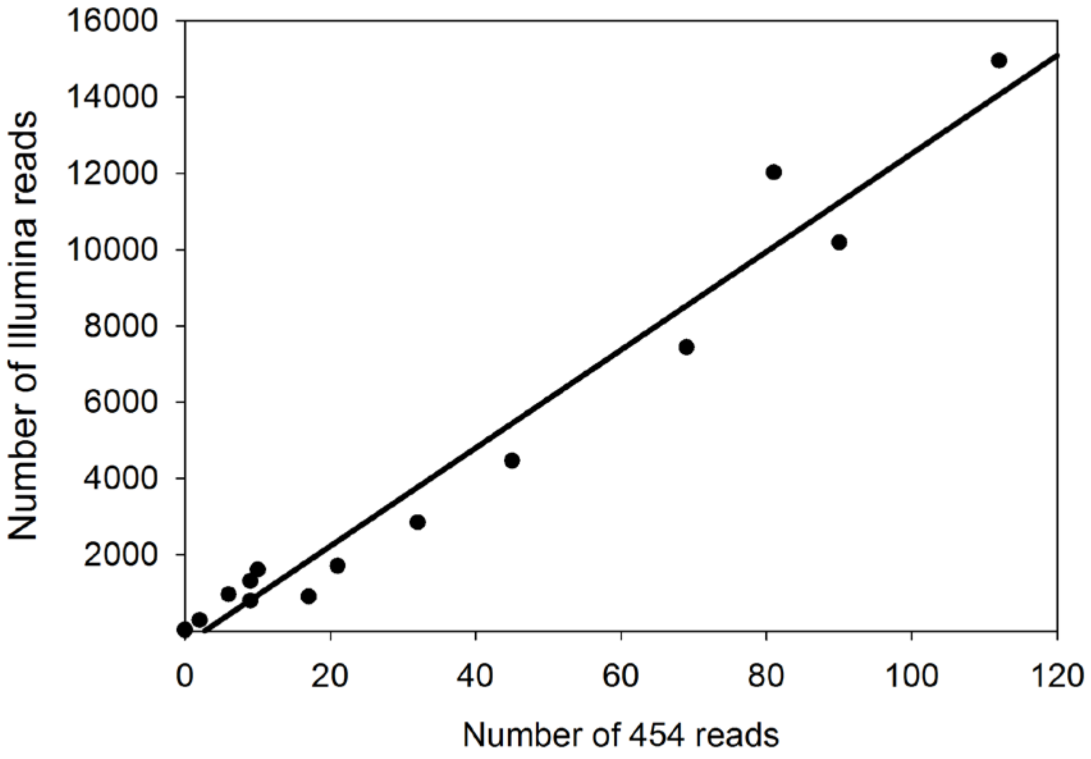

Supplementary Table S1 A – G Top 25 OTUs detected in each sample based on % of total reads in 16S rRNA gene sequencing analysis, and the best BLAST hit to a cultured reference strain (% identity in final column).

Supplementary Table S1 A Best BLAST hits to the top OTUs detected in an analysis of 16S rRNA amplicons from OSPW sampled in August 2011.

Rank	% Reads	Phylum	Class	Order	Family	Genus	% Identity
1	4.66	*Proteobacteria*	*Betaproteobacteria*	*Burkholderiales*	*Comamonadaceae*	*Hydrogenophaga*	99
2	3.88	*Bacteroidetes*	*Flavobacteria*	*Flavobacteriales*	*Flavobacteriaceae*	*Lutibacter*	98
3	3.38	*Proteobacteria*	*Gammaproteobacteria*	*Pseudomonadales*	*Moraxellaceae*	*Enhydrobacter*	99
4	2.99	*Proteobacteria*	*Betaproteobacteria*	*Burkholderiales*	*Burkholderiaceae*	*Limnobacter*	99
5	2.99	*Proteobacteria*	*Gammaproteobacteria*	*Methylococcales*	*Methylococcaceae*	*Methylogaea*	94
6	2.89	*Bacteroidetes*	*Saprospiraceae*	*Sphingobacteriales*	*Sphingobacteriia*	*Haliscomenobacter*	93
7	2.59	*Bacteroidetes*	*Bacteroidia*	*Bacteroidales*	*Porphyromonadaceae*	*Barnesiella*	90
8	2.59	*Bacteroidetes*	*Flavobacteriia*	*Flavobacteriales*	*Cryomorphaceae*	*Fluviicola*	96
9	2.39	*Bacteroidetes*	*Flavobacteria*	*Flavobacteriales*	*Flavobacteriaceae*	*Flavobacterium*	98
10	2.29	*Proteobacteria*	*Alphaproteobacteria*	*Rhizobiales*	*Xanthobacteraceae*	*Xanthobacter*	99
11	2.19	*Proteobacteria*	*Betaproteobacteria*	*Burkholderiales*	*Burkholderiaceae*	*Sphaerotilus*	99
12	1.89	*Bacteroidetes*	*Sphingobacteriia*	*Sphingobacteriales*	*Chitinophagaceae*	*Balneola*	89
13	1.79	*Proteobacteria*	*Betaproteobacteria*	*Rhodocyclales*	*Rhodocyclaceae*	*Rhodocyclus*	96
14	1.59	*Planctomycetes*	*Planctomycetia*	*Planctomycetales*	*Planctomycetaceae*	*Roseimaritima*	93
15	1.39	*Proteobacteria*	*Betaproteobacteria*	*Burkholderiales*	*Comamonadaceae*	*Variovorax*	99
16	1.39	*Proteobacteria*	*Alphaproteobacteria*	*Rhizobiales*	*Bradyrhizobiaceae*	*Variibacter*	94
17	1.29	*Planctomycetes*	*Planctomycetia*	*Planctomycetales*	*Isosphaeraceae*	*Aquisphaera*	90
18	1.19	*Proteobacteria*	*Betaproteobacteria*	*Burkholderiales*	*Alcaligenaceae*	*Achromobacter*	96
19	1.19	*Bacteroidetes*	*Flavobacteria*	*Flavobacteriales*	*Flavobacteriaceae*	*Flavobacterium*	98
20	1.09	*Tenericutes*	*Mollicutes*	*Acholeplasmatales*	*Acholeplasmataceae*	*Acholeplasma*	95

Supplementary Table S1 B Best BLAST hits to the top OTUs detected in an analysis of 16S rRNA amplicons from heavy SIP fraction (fraction 5) of OSPW sampled in August, 2011.

Rank	% Reads	Phylum	Class	Order	Family	Genus	% Identity
1	23.31	*Proteobacteria*	*Alphaproteobacteria*	*Rhizobiales*	*Hyphomicrobiaceae*	*Devosia*	96
2	12.64	*Proteobacteria*	*Gammaproteobacteria*	*Methylococcales*	*Methylococcaceae*	*Methylomonas*	96
3	9.03	*Proteobacteria*	*Betaproteobacteria*	*Rhodocyclales*	*Rhodocyclaceae*	*Thauera*	99
4	8.53	*Proteobacteria*	*Alphaproteobacteria*	*Caulobacterales*	*Caulobacteraceae*	*Brevundimonas*	100
5	8.29	*Proteobacteria*	*Gammaproteobacteria*	*Methylococcales*	*Methylococcaceae*	*Methylogaea*	94
6	6.79	*Planctomycetes*	*Planctomycetia*	*Planctomycetales*	*Planctomycetaceae*	*Thermogutta*	87
7	4.95	*Proteobacteria*	*Gammaproteobacteria*	*Methylococcales*	*Methylococcaceae*	*Methylomicrobium*	99
8	4.52	*Proteobacteria*	*Alphaproteobacteria*	*Magnetococcales*	*Magnetococcaceae*	*Magnetococcus*	90
9	4.25	*Proteobacteria*	*Alphaproteobacteria*	*Rhodospirillales*	*Acetobacteraceae*	*Acidiphilium*	99
10	4.21	*Proteobacteria*	*Betaproteobacteria*	*Rhodocyclales*	*Rhodocyclaceae*	*Methyloversatilis*	100
11	3.28	*Euryarchaeota*	*Methanomicrobia*	*Methanomicrobiales*	*Methanoregulaceae*	*Methanoregula*	99
12	2.98	*Planctomycetes*	*Planctomycetia*	*Planctomycetales*	*Planctomycetaceae*	*Thermogutta*	86
13	2.88	*Proteobacteria*	*Betaproteobacteria*	*Burkholderiales*	*Comamonadaceae*	*Hydrogenophaga*	99
14	2.47	*Proteobacteria*	*Gammaproteobacteria*	*Xanthomonadales*	*Algiphilaceae*	*Algiphilus*	94
15	2.34	*Proteobacteria*	*Alphaproteobacteria*	*Sphingomonadales*	*Erythrobacteraceae*	*Altererythrobacter*	99
16	2.21	*Euryarchaeota*	*Methanomicrobia*	*Magnetococcales*			99
17	2.17	*Euryarchaeota*	*Methanomicrobia*	*Methanosarcinales*	*Methanosaetaceae*	*Methanosaeta*	100
18	1.97	*Proteobacteria*	*Alphaproteobacteria*	*Sphingomonadales*	*Erythrobacteraceae*	*Porphyrobacter*	100
19	1.81	*Proteobacteria*	*Gammaproteobacteria*	*Chromatiales*	*Chromatiaceae*	*Thiococcus*	95
20	1.81	*Proteobacteria*	*Alphaproteobacteria*	*Rhodocyclales*	*Rhodocyclaceae*	*Azospirillum*	93

Supplementary Table S1 C Best BLAST hits to the top OTUs detected in an analysis of 16S rRNA amplicons from heavy SIP Fractions (fractions 4 and 5) of OSPW sampled in August, 2011, amended with labeled benzene for 9 days.

Rank	% Reads	Phylum	Class	Order	Family	Genus	% Identity
1	86.87	*Proteobacteria*	*Betaproteobacteria*	*Rhodocyclales*	*Rhodocyclaceae*	*Methyloversatilis*	100
2	5.18	*Proteobacteria*	*Alphaproteobacteria*	*Rhodospirillales*	*Acetobacteraceae*	*Zavarzinia*	99
3	1.39	*Proteobacteria*	*Gammaproteobacteria*	*Xanthomonadales*	*Algiphilaceae*	*Algiphilus*	94
4	0.62	*Proteobacteria*	*Deltaproteobacteria*	*Syntrophobacterales*	*Syntrophobacteraceae*	*Desulforhabdus*	90
5	0.51	*Proteobacteria*	*Deltaproteobacteria*	*Desulfuromonadales*	*Geobacteraceae*	*Geobacter*	91
6	0.42	*Proteobacteria*	*Betaproteobacteria*	*Rhodocyclales*	*Rhodocyclaceae*	*Rhodocyclus*	96
7	0.24	*Proteobacteria*	*Betaproteobacteria*	*Rhodocyclales*	*Rhodocyclaceae*	*Dechloromonas*	94
8	0.23	*Proteobacteria*	*Alphaproteobacteria*	*Rhizobiales*	*Bradyrhizobiaceae*	*Bradyrhizobium*	95
9	0.19	*Chloroflexi*	*Anaerolineae*	*Anaerolineales*	*Anaerolineaceae*	*Bellilinea*	92
10	0.17	*Proteobacteria*	*Gammaproteobacteria*	*Chromatiales*	*Chromatiaceae*	*Thiococcus*	95
11	0.17	*Planctomycetes*	*Planctomycetia*	*Planctomycetales*	*Planctomycetaceae*	*Gemmata*	91
12	0.16	*Planctomycetes*	*Planctomycetia*	*Planctomycetales*	*Planctomycetaceae*	*Thermogutta*	86
13	0.13	*Proteobacteria*	*Alphaproteobacteria*	*Rhodospirillales*	*Rhodospirillaceae*	*Inquilinus*	94
14	0.11	*Tenericutes*	*Mollicutes*	*Entomoplasmatales*	*Spiroplasmataceae*	*Spiroplasma*	75
15	0.09	*Proteobacteria*	*Betaproteobacteria*	*Rhodocyclales*	*Rhodocyclaceae*	*Sulfuritalea*	99
16	0.08	*Proteobacteria*	*Deltaproteobacteria*	*Desulfuromonadales*	*Desulfuromonadaceae*	*Desulfuromusa*	90
17	0.07	*Acidobacteria*	*Solibacteres*	*Solibacterales*	*Solibacterales*	*Bryobacter*	93
18	0.07	*Proteobacteria*	*Betaproteobacteria*	*Burkholderiales*	*Comamonadaceae*	*Caenimonas*	98
19	0.07	*Proteobacteria*	*Alphaproteobacteria*	*Sphingomonadales*	*Erythrobacteraceae*	*Altererythrobacter*	99
20	0.06	*Proteobacteria*	*Deltaproteobacteria*	*Desulfobacterales*	*Desulfobacteraceae*	*Desulfotignum*	85

Supplementary Table S1 D Best BLAST hits to the top OTUs detected in an analysis of 16S rRNA amplicons from heavy SIP Fractions (fractions 5 and 6) of OSPW sampled in August, 2011, amended with labeled naphthalene for 7 days.

Rank	% Reads	Phylum	Class	Order	Family	Genus	% Identity
1	45.56	*Proteobacteria*	*Gammaproteobacteria*	*Chromatiales*	*Chromatiaceae*	*Thiococcus*	95
2	20.50	*Proteobacteria*	*Betaproteobacteria*	*Rhodocyclales*	*Rhodocyclaceae*	*Thauera*	99
3	8.19	*Proteobacteria*	*Gammaproteobacteria*	*Pseudomonadales*	*Pseudomonadaceae*	*Pseudomonas*	100
4	2.69	*Proteobacteria*	*Gammaproteobacteria*	*Pseudomonadales*	*Pseudomonadaceae*	*Pseudomonas*	96
5	5.73	*Proteobacteria*	*Gammaproteobacteria*	*Pseudomonadales*	*Pseudomonadaceae*	*Pseudomonas*	98
6	1.61	*Proteobacteria*	*Betaproteobacteria*	*Burkholderiales*	*Comamonadaceae*	*Acidovorax*	99
7	1.24	*Proteobacteria*	*Gammaproteobacteria*	*Pseudomonadales*	*Pseudomonadaceae*	*Pseudomonas*	97
8	1.23	*Proteobacteria*	*Alphaproteobacteria*	*Rhodobacterales*	*Rhodobacteraceae*	*Gemmobacter*	99
9	0.73	*Proteobacteria*	*Gammaproteobacteria*	*Pseudomonadales*	*Pseudomonadaceae*	*Pseudomonas*	98
10	0.57	*Chloroflexi*	*Caldilineae*	*Caldilineales*	*Caldilineaceae*	*Caldilinea*	83
11	0.39	*Proteobacteria*	*Betaproteobacteria*	*Rhodocyclales*	*Rhodobacteraceae*	*Azoarcus*	99
12	0.39	*Proteobacteria*	*Gammaproteobacteria*	*Pseudomonadales*	*Pseudomonadaceae*	*Azotobacter*	100
13	0.39	*Proteobacteria*	*Gammaproteobacteria*	*Pseudomonadales*	*Pseudomonadaceae*	*Pseudomonas*	98
14	0.35	*Proteobacteria*	*Alphaproteobacteria*	*Sphingomonadales*	*Sphingomonadaceae*	*Novosphingobium*	98
15	0.34	*Proteobacteria*	*Gammaproteobacteria*	*Pseudomonadales*	*Pseudomonadaceae*	*Pseudomonas*	98
16	0.30	*Proteobacteria*	*Alphaproteobacteria*	*Sphingomonadales*	*Erythrobacteraceae*	*Porphyrobacter*	100
17	0.28	*Proteobacteria*	*Gammaproteobacteria*	*Legionellales*	*Coxiellaceae*	*Aquicella*	95
18	0.24	*Proteobacteria*	*Gammaproteobacteria*	*Pseudomonadales*	*Pseudomonadaceae*	*Pseudomonas*	99
19	0.23	*Proteobacteria*	*Alphaproteobacteria*	*Caulobacterales*	*Caulobacteraceae*	*Phenylobacterium*	97
20	0.19	*Proteobacteria*	*Gammaproteobacteria*	*Pseudomonadales*	*Pseudomonadaceae*	*Pseudomonas*	98

Supplementary Table S2 Best BLAST hits to the top OTUs detected in an analysis of 16S rRNA amplicons from heavy SIP Fractions of OSPW sampled in August 2011, amended with labeled benzene for 14 days.

Rank	% Reads	Phylum	Class	Order	Family	Genus
1	43.85	*Proteobacteria*	*Betaproteobacteria*	*Rhodocyclales*	*Rhodocyclaceae*	*Methyloversatilis*
2	19.58	*Proteobacteria*	*Alphaproteobacteria*	*Caulobacterales*	*Caulobacteraceae*	*Brevundimonas*
3	8.04	*Proteobacteria*	*Betaproteobacteria*	*Burkholderiales*	*Comamonadaceae*	*Diaphorobacter*
4	6.04	*Planctomycetes*	*Planctomycea*	*Planctomycetales*	*Planctomycetaceae*	*Planctomyces*
5	2.91	*Proteobacteria*	*Alphaproteobacteria*	*Sphingomonadales*	*Erythrobacteraceae*	*Erythromicrobium*
6	2.73	*Proteobacteria*	*Betaproteobacteria*	*Burkholderiales*	*Comamonadaceae*	
7	1.19	*Proteobacteria*	*Betaproteobacteria*	*Rhodocyclales*	*Rhodocyclaceae*	*Thauera*
8	0.79	*Proteobacteria*	*Alphaproteobacteria*	*Rhodobacterales*	*Hyphomonadaceae*	
9	0.63	*Proteobacteria*	*Alphaproteobacteria*	*Rhizobiales*	*Hyphomicrobiaceae*	*Rhodoplanes*
10	0.61	*Proteobacteria*	*Betaproteobacteria*	*Rhodocyclales*	*Rhodocyclaceae*	*Methyloversatilis*
11	0.57	*Proteobacteria*	*Alphaproteobacteria*	*Caulobacterales*	*Caulobacteraceae*	*Brevundimonas*
12	0.57	*Proteobacteria*	*Betaproteobacteria*	*Rhodocyclales*	*Rhodocyclaceae*	*Methyloversatilis*
13	0.55	*Planctomycetes*	*Phycisphaerae*	*Phycisphaerae*	*Phycisphaerales*	
14	0.55	*Proteobacteria*	*Alphaproteobacteria*	*Caulobacterales*	*Caulobacteraceae*	*Caulobacte*
15	0.53	*Proteobacteria*	*Alphaproteobacteria*	*Rhodospirillales*	*Rhodospirillacea*	
16	0.51	*Proteobacteria*	*Betaproteobacteria*	*Rhodocyclales*	*Rhodocyclaceae*	*Methyloversatilis*
17	0.48	*Proteobacteria*	*Gammaproteobacteria*	*Xanthomonadales*	*Xanthomonadacea*	*Dokdonella*
18	0.46	*Proteobacteria*	*Alphaproteobacteria*	*Rhizobiales*	*Phyllobacteriaceae*	*Mesorhizobium*
19	0.40	*Proteobacteria*	*Alphaproteobacteria*	*Rhodobacterales*	*Hyphomonadaceae*	*Hyphomonas*
20	0.38	*Proteobacteria*	*Alphaproteobacteria*	*Rhodospirillales*	*Rhodospirillaceae*	

Supplementary Table S3 Metagenome features of surface WIP-OSPW sampled in August 2011 of the combined Illumina and 454 assembly obtained from the IMG pipeline.

	Assembled	
	**Number**	**% of Assembled**
**Number of sequences**	1,520,282	100.00%
**Number of bases**	728,742,540	100.00%
GC count	426,300,665	58.50%
**Genes**		
RNA genes	11,467	0.63%
rRNA genes	2,282	0.13%
5S rRNA	396	0.02%
16S rRNA	632	0.03%
18S rRNA	38	0.00%
23S rRNA	1,170	0.06%
28S rRNA	46	0.00%
tRNA genes	9,185	0.50%
Protein coding genes	1,811,780	99.37%
with Product Name	852,731	46.77%
with COG	1,088,850	59.72%
with Pfam	961,630	52.74%
with KO	873,050	47.88%
with Enzyme	511,566	28.06%
with MetaCyc	360,503	19.77%
with KEGG	520,901	28.57%
**COG Clusters**	4,559	98.45%
**Pfam Clusters**	6,650	40.81%
**Biosynthetic Clusters**	554	
Genes in Biosynthetic Clusters	2,711	

Supplementary Table S4 List of bacterial isolates obtained from various OSPW enrichments. The identifications are based on BLAST analysis of nearly complete 16S rRNA gene sequences.

**Class**	**Order**	**Family**	**Genus**	**GenBank taxonomy**	**Source/Medium of isolation**	**Sample(s) where OTUs were found**	**Query length (bp)**	**% Identity**	**Strain name**	**Growth on benzene**	**Growth of naphtha-lene**
Alphaproteobacteria	*Rhizobiales*	*Xanthobacteraceae*	*Xanthobacter*	*Xanthobacter tagetidis*	20R2A-T	C, M	1205	97	OSP W1	+	-
Alphaproteobacteria	*Rhodospirillales*	*Acetobacteraceae*	*Zavarzinia*	*Zavarzinia compransoris*	20R2A-T	B	1355	99	OSP W2	+	-
Gammaproteobacteria	*Pseudomonadales*	*Pseudomonadaceae*	*Pseudomonas*	*Pseudomonas stutzeri*	M10-T with naphthalene vapour	N, OSPW	1374	99	OSP W3	-	+
Betaproteobacteria	*Rhodocyclales*	*Rhodocyclaceae*	*Thauera*	*Thauera phenylacetica*	20R2A-T with naphthalene vapour	N, C, OSPW	1387	99	OSP W4	-	?

B=benzene SIP heavy fraction, N=naphthalene SIP heavy fraction, M= methanol SIP heavy fraction, C= control (unamended OSPW) SIP heavy fraction, OSPW=fresh OSPW sample
